# CBMR: Coordinate-based meta-regression for group and covariate inference

**DOI:** 10.1162/IMAG.a.1057

**Published:** 2025-12-19

**Authors:** Yifan Yu, Lauren D. Hill-Bowen, Michael Cody Riedel, Katherine Bottenhorn, Angela R. Laird, Thomas E. Nichols

**Affiliations:** Oxford Big Data Institute, University of Oxford, Oxford, United Kingdom; Department of Psychiatry and Behavioral Sciences, Vanderbilt University Medical Center, Nashville, TN, United States; Department of Physics, Florida International University, Miami, FL, United States; Department of Population and Public Health Sciences, University of Southern California, Los Angeles, CA, United States; Center for Imaging Science, Florida International University, Miami, FL, United States; Nuffield Department of Clinical Neurosciences, Wellcome Centre for Integrative Neuroimaging, FMRIB Oxford, United Kingdom

**Keywords:** neuroimaging data, coordinate-based meta-analysis, generalised linear models, spatial statistics, statistical modelling

## Abstract

Coordinate-based meta-analysis synthesises findings from multiple neuroimaging studies to identify consistent patterns of brain activation. However, comparing foci distributions between groups of studies remains a challenge, usually requiring balanced sample sizes. In this work, we introduce a multi-group coordinate-based meta-regression (CBMR) framework that employs a generative spline-based spatial model regularised by a roughness penalty, providing flexible control over smoothness. We conduct extensive evaluations with simulations and demonstrate the method on real data. We find that when all groups have at least 200 foci, parametric inference is valid, while sparser datasets require inference via parametric bootstrap. This work is implemented and freely available as a module within the Python package NiMARE, enabling flexible meta-regression and inference for coordinate-based meta-analytic functional MRI datasets involving multiple groups.

## 1 Introduction

### 1.1 Background

Functional neuroimaging infers brain activity by monitoring fluctuations in cerebral blood flow, oxygen consumption, or metabolic processes. These techniques include, for example, positron emission tomography (PET) and functional MRI (fMRI). The field of fMRI, in particular, has experienced significant improvements, with substantial growth in the literature on brain activations and an increasingly common practice of sharing findings publicly. For example, BrainMap (Laird et al., 2005) and Neurosynth (Yarkoni et al., 2011) are large-scale neuroimaging databases containing brain activation data from 22, 504 and 14, 371 studies, respectively. These developments motivate the need to aggregate and synthesise findings from multiple independent but related fMRI studies to understand consistency and heterogeneity, and improve the precision of inferences about brain activations associated with cognitive tasks. Analysing activations from a single fMRI study is often unreliable and lacks sufficient statistical power due to common limitations, such as small sample sizes, high prevalence of false positives (e.g., Wager et al. (2007) found approximately 10−20% of reported foci in publications are false positives), significant heterogeneity among studies, and unreliable inference caused by variability in measurements and analysis methods (Samartsidis et al., 2017). Meta-analysis is an essential tool for addressing these limitations, improving statistical power by pooling evidence from multiple studies to produce more consistent and reliable findings. While this work focuses exclusively on task fMRI data, this approach could potentially extend to other types of neuroimaging data, such as resting-state fMRI and structural analyses using voxel-based morphometry.

Meta-analyses in neuroimaging research can be either imaged based or coordinate based. Image-based meta-analysis (IBMA) utilises 3D statistical maps from original studies, while coordinate-based meta-analysis (CBMA) only includes reported spatial locations of activation foci in standard MNI or Talairach space. IBMA is considered the ideal approach, as full statistical maps preserve both the locations and intensities of activation regions. In contrast, CBMA experiences substantial information loss, as it only reports the locations of activation foci (local maxima), typically fewer than 10 foci per study, and often ignores deactivations (Salimi-Khorshidi et al., 2009). Despite these limitations, CBMA has historically remained the predominant method, largely due to the challenges of storing and sharing full statistical maps, as highlighted by a recent survey of software and methods used in fMRI meta-analyses published between 2019 and 2024 (Yeung, 2025). However, the practice of sharing entire statistical maps has become increasingly common in recent years, and the development of software and platforms (e.g., NiMARE; Salo et al., 2022) provides a modular and unified interface that facilitates the adoption of more comprehensive meta-analytic approaches.

Within CBMA methods, researchers have developed various kernel-based or coordinate-based approaches. The key distinction between these approaches lies in whether they use spatial kernel functions to model activation patterns around each reported focus or statistical models to estimate underlying brain functions. For instance, activation likelihood estimation (ALE; Eickhoff et al., 2012) uses a Gaussian kernel, multilevel kernel density analysis (MKDA; Wager et al., 2007) employs a uniform sphere, and signed differential mapping (SDM; Radua et al., 2012) applies a Gaussian kernel scaled by effect size. These kernel-based methods derive statistical inferences by referencing a null hypothesis of random foci arrangement (Samartsidis et al., 2017). The Benjamini–Hochberg (BH) procedure is commonly applied to control the family-wise error rate (FWE) or the false discovery rate (FDR) in multiple testing corrected inferences (Benjamini & Hochberg, 1995). However, kernel-based CBMA approaches have been criticised for several limitations, including lack of interpretability, difficulty in group comparisons among imbalanced groups, inability to model the spatial dependence of activation foci, and challenges in incorporating study-level covariates for meta-regression (Samartsidis et al., 2019).

Bayesian model-based methods have addressed these limitations by introducing either parametric spatial point process models (Kang et al., 2011; Montagna et al., 2018; Samartsidis et al., 2019) or non-parametric Bayesian models (Kang et al., 2014; Yue et al., 2012). These approaches are based on explicit generative models with testable assumptions. While Bayesian model-based methods are generally more accurate and interpretable than kernel-based approaches, they are also more computationally intensive, often requiring parallel computation on GPUs (Samartsidis et al., 2019). Moreover, some of these methods are unable to perform meta-regression to estimate the effect of study-level covariates, limiting their ability to evaluate how these covariates globally influence spatial activation intensity functions specific to each study.

A comparative summary of the advantages and disadvantages of IBMA and CBMA methods is provided in Table 1. To address the limitations of both kernel-based and Bayesian model-based approaches, we propose a classical frequentist meta-regression framework that explicitly captures the spatial structure of the activation foci distribution (Yu et al., 2024). This model is formulated as a generalised linear model (GLM) and consists of two key components: a spatial effect, which incorporates a spline parameterisation to generate a smooth response across the entire brain image; and a global effect to account for study-level covariates specific to each study. Four different stochastic models within the GLM framework have been considered: While Poisson is the classic distribution for approximating foci distribution (as a low-rate Binomial distribution) at the voxel level, Samartsidis et al. (2020) have found evidence of over-dispersion in CBMA data. To address this, we further explore a Negative Binomial model, a Clustered Negative model, and a Quasi-Poisson model to account for the excess variation in foci data. However, there are practical challenges in the implementation and optimisation of this meta-regression approach for real fMRI datasets. With fewer than 10 reported foci per study on average, the values of spatial regressors become highly negative during optimisation. This leads to difficulties in convergence and poses challenges for statistical inference, particularly when estimating the covariance structure between different voxels. In this work, we demonstrate that applying a roughness penalty to the spatial spline parameterisation improves the numerical stability of the meta-regression and enhances the precision of statistical inference. This modification also enables the estimation of group-wise intensity functions for multiple groups, and facilitates group comparisons for spatial activation intensity. By introducing this penalty, we overcome the strict limitation imposed by the minimum number of foci per group for meta-regression, making comparisons across multiple groups possible.

**Table 1.** Comparison of the strengths and weaknesses of existing IBMA and CBMA methods relative to the proposed CBMR approach.

METHOD	Strength	Weakness
IBMA (Image-based meta-analysis)	• Uses full 3D statistical maps (locations + intensities) • Preserves full spatial information, including deactivations • Enables statistical modelling • Less bias from reporting practices	• Requires access to full unthresholded statistical maps (not widely shared) • Data storage and sharing burden • Limited by inconsistent preprocessing across studies
CBMA (Kernel-Based:ALE, MKDA, SDM)	• Widely used and historically dominant • Requires only reported peak coordinates (easy to extract) • Computationally efficient • Simple to implement and interpret	• Severe information loss • Kernel choice is arbitrary and may bias results • Limited ability for unbalanced group comparisons • Ignore spatial dependence
CBMA (Model-based: Parametric spatial point process/non-parametric Bayesian models)	• Explicit generative statistical framework • Improved interpretability • Capture spatial dependence of foci • Greater accuracy • Flexible Bayesian framework allows uncertainty quantification	• High computational demand (often requires parallel or GPU resources) • Slower inference compared with kernel-based CBMA • Challenging to scale to thousands of studies
CBMR (Coordinate-based meta-regression)	• Explicit spatial statistical model with basis functions • Incorporates study-level covariates • Allows (imbalanced) group comparisons • Improves interpretability and inference validity	• Computationally more intensive than kernel-based CBMA • Requires bootstrapping inference for dataset with insufficient number of foci

A key limitation of our previous meta-regression framework is its restriction to a single group of studies, although this approach is consistent with the neuroimaging meta-analysis literature that often aggregates heterogeneous studies into a single group (Eickhoff et al., 2009; Salo et al., 2022). In practice, however, investigators are often interested in comparing distinct collections of studies rather than collapsing them into one group. Typical scenarios include (1) contrasting study collections of different patient populations versus those of healthy controls to separate disease-related from normative activation patterns (Caspersen et al., 2021); (2) comparing sets of studies using different task variants (e.g., 1-back-vs-0-back vs. 2-back-vs-0-back working-memory studies) to test whether increasing cognitive load systematically modulates canonical networks (Emch et al., 2019; Owen et al., 2005); (3) contrasting studies with different stimulus modalities (visual vs. auditory language tasks) to localise modality-specific activation patterns (Price, 2012); (4) comparing collections of studies using different pain paradigms (e.g., thermal vs. mechanical pain) to identify overlapping and distinct neural circuits in modality-dependent pain processing (Lancaster et al., 2012; Wiech, 2016); and (5) multi-dataset analyses where site, scanner, or acquisition acts as a grouping factor. Single-group CBMA methods cannot estimate group-specific activation maps or statistically test group differences in activation probability (Biswal et al., 2010). A multi-group CBMR framework addresses these limitations by jointly estimating group-level activation maps, and enabling direct group contrasts, while stabilising group-specific estimation with our spatial basis approach and allowing parsimonious modelling of global study-specific effects (e.g., sample size, publication year).

A further limitation of our previous meta-regression framework was that the covariance structure of spatial intensity across different voxel locations, estimated from the inverse Fisher information, could encounter numerical issues in small datasets, leading to underestimation of covariance. To address this, we replaced the parametric inference based on the Wald test with parametric and non-parametric bootstrapping approaches, allowing for tests of spatial homogeneity within each group and group equality in multi-group datasets. Additionally, we explored parallelising code execution to accelerate the bootstrapping process, making it a computationally feasible alternative to the traditional parametric Wald test. We then demonstrate the validity of bootstrap-based statistical inference on both simulated and real datasets, comparing its activation maps with that of traditional kernel-based methods.

In this paper, we present a coordinate-based meta regression and inference (CBMR) framework for multiple groups, a Python-based tool that allows for the estimation of both group-specific spatial regressors and regressors for study-level covariates, as well as statistical inference for spatial homogeneity and equality of group-specific intensity functions. The CBMR tool is integrated into the Python package NiMARE (Salo et al., 2022), and will be accessible through a web-based platform, Neurosynth Compose. This platform allows customised neuroimaging meta-analyses using either self-uploaded data or data imported directly from the Neurosynth database, providing a wide range of CBMA methods with no programming experience required. Our current implementation of the CBMR framework consists of meta-regression and meta-inference modules. The meta-regression module can be executed independently to estimate group-specific intensity functions, while the meta-inference module uses the optimised regressors from the meta-regression module as input and supports flexible (single or multiple, independent or simultaneous) hypothesis testing on either spatial homogeneity or group equality, which can be easily specified with a contrast matrix.

In the following sections, we first provide background on spline parameterisation for modelling spatial dependence, as well as the stochastic models, parameter estimation, and inferences in CBMR. Following this, we give preliminary statistical information describing the single-group CBMR and its extension to multi-group settings. In the Method section, we outline the computational pipeline of CBMR, starting with input specification and dimension reduction, followed by parallelised execution of optimisation, parameter estimation and finally, inference using either the parametric Wald test or a bootstrapping approach. Next, we evaluate the validity and performance of CBMR through simulations and comparisons with existing kernel-based and model-based approach on real datasets. Finally, we conclude with a real dataset example of cue-reactivity task.

#### 1.1.1 Spatial model: Spline parameterisation

Gaussian and uniform kernels are commonly used in kernel-based CBMA methods to model the spatial distribution of reported foci, smoothing, and estimating the probability of activation around each focus to capture spatial uncertainty in neuroimaging data effectively. In contrast, model-based CBMA methods have previously treated each study’s foci as realisations of a doubly-stochastic Poisson process, also known as a Cox process, in spatial point process modelling of CBMA data. In some of these model-based approaches, the log intensity function is parametrised either by superimposed Gaussian kernel basis functions or as a Gaussian process (Montagna et al., 2018; Samartsidis et al., 2019). These previous studies highlight the importance of applying spatial models to explain spatial uncertainty in neuroimaging data.

Here, we propose a spatial model parametrised by a tensor product of cubic B-spline basis functions. This spatial basis is chosen for its smoothness, stability, and flexibility, as the level of spatial smoothness is parametrised by knots spacing: larger knots spacing generates fewer basis functions and thus greater smoothness, while closer knots produce more basis functions and enhance the model’s ability to capture fine details. After setting the knot spacing uniformly across the x, y, and z directions, we construct a B-spline curve as a linear combination of the B-spline basis functions in each direction. We then evaluate the coefficients at each voxel corresponding to the B-spline bases to construct a coefficient matrix for each direction. The three-dimensional coefficient matrix of B-spline bases is then constructed by taking the tensor product of the three coefficient matrices along each of the x,y,z directions, further details are outlined in Yu et al. (2024).

We assert that the spatial model parametrised by spline bases is capable of efficiently capturing spatial uncertainty. This is supported by both its demonstrated effectiveness in previous experiments within single-group CBMR settings and comparison with alternative spatial models, such as Gaussian kernels. Minimal differences were observed between these two spatial models in both simulated and real datasets, as detailed in Yu et al. (2024). Accordingly, we believe it is reasonable to adopt this spatial model in the current work.

#### 1.1.2 CBMR parameter estimation

A vast amount of literature exists on the development of tools and methodologies for generalised linear models (GLM). Since the formalisation of GLMs in 1972 (Nelder & Wedderburn, 1972), iterative re-weighted least squares (IRLS) have been recognised as a reliable and efficient computational approach for parameter estimation, effectively addressing the complexity introduced by the non-linear relationships. IRLS became the standard method for parameter estimation in GLMs. Later, the Newton–Raphson method and its variation, Fisher scoring, were proposed and widely adopted due to their faster convergence and improved efficiency and numerical stability, particularly on highly non-linear optimisation surfaces (Jennrich & Sampson, 1976). Since the 1990s, regularised estimation methods that add a penalty term (e.g., Lasso, Ridge, and Elastic Net) to the likelihood function have also been developed, encouraging sparsity and stability in parameter estimation (Hoerl & Kennard, 1970; Tibshirani, 1996; Zou & Hastie, 2005). More recently, several tools and software built upon these foundational methods have been developed for GLMs parameter estimation. Among the most popular are R packages such as *glmnet* (Friedman et al., 2010), *MASS* (Venables & Ripley, 2002), and *lme4* (Bates, 2014), as well as Python packages *statsmodels* (Seabold & Perktold, 2010), which have made GLM parameter estimation accessible and scalable, supporting MLE, IRLS, and Bayesian methods. These tools, along with advancements in computing, enable efficient parameter estimation for GLMs, even with large datasets and complex models.

However, in meta-regression of fMRI data, parameter estimation is performed for a model with two components: the spatial effect which includes hundreds of thousands of different voxels within the brain mask for each study, and the global effect of study-level covariates which moderates the intensity function of a specific study by a constant. For a large scale, voxel-wise GLM analysis to fully optimise the computational efficiency, it is essential to vectorise computation across voxels. Many existing GLM tools and software are developed with operations that are not fully vectorised, especially when dealing with complex or large-scale data structures. Handling high-dimensional data across iterative computations without careful memory management can limit vectorisation, and GLMs applied to sparse or irregular data further complicate vectorisation due to the challenges brought by sparse matrices. While for likelihood functions with regularisation terms (e.g., Lasso, Ridge, Elastic Net), additional iterative processes such as coordinate descent are required (Friedman et al., 2010), making it even more difficult to fully vectorise and parallelise these computations. Operations that are not amenable to vectorisation create bottlenecks in large-scale GLM optimisation, as they must be executed separately for each voxel in each study, significantly slowing down computation. As a result, many existing software for GLMs analysis is not suitable for large-scale or complex CBMA data.

Additionally, we believe that efforts should focus on reducing the dimensionality of the variables rather than the combined product of the number of studies and voxels used as dimensions. We provide rigorous proofs demonstrating that the GLM with various stochastic models can be simplified to equivalent forms with sufficient statistics, with dimensions no greater than either the number of voxels or the number of studies (Yu et al., 2024). We will continue to follow this approach in the current work.

As an efficient and fundamental approach for parameter estimation in GLMs, Maximum Likelihood Estimation (MLE) is widely used to optimise the model by maximising the probability of the observed data given a set of parameters, under the assumptions of the GLM. One effective optimisation algorithm for this task is the Limited-memory Broyden–Fletcher–Goldfarb–Shanno (L-BFGS), a quasi-Newton method known for its memory and computational efficiency in handling high-dimensional data and parameters, as it approximates the Hessian matrix rather than computing it explicitly. L-BFGS is also chosen for its faster convergence compared with simpler gradient descent methods, especially in scenarios with complex or irregular likelihood curvatures (Liu & Nocedal, 1989). We have observed its effectiveness in optimising single-group CBMR scenarios (Yu et al., 2024), and we will extend it to the current work, a more complex multi-group CBMR setting.

#### 1.1.3 Inference

For CBMA, the central object of interest is to identify the brain activation regions associated with a specific cognitive task, or to find differences in activation regions in response to similar but distinct stimuli. This requires fMRI GLM analyses to conclude with significance-based hypothesis tests, conducting either homogeneity tests or group comparison tests at the voxel-wise level using Wald test statistics. The Wald-based hypothesis testing procedures in the neuroimaging applications include tests on both single and multiple parameters. The single-parameter test assesses whether the estimated intensity at each voxel is greater than the average intensity expected under a random distribution of foci, while the multiple-parameter test evaluates whether a specific linear combination of group-wise estimated intensities is distinguishable from zero at each voxel. In some circumstances, it may also be of practical interest to assess multiple flexible group comparison hypotheses simultaneously. As the effect of study-level covariates is an additional component of CBMR, single- or multiple-parameter hypothesis testing is also applicable to these covariates. This allows for assessment of whether a specific study-level covariate has a significant effect or whether multiple study-level covariates have equivalent effects in CBMR, following the same Wald test procedure.

In existing GLM tools and software, Wald tests are commonly implemented to assess the significance of individual coefficients or groups of coefficients, typically for testing whether they are significantly different from zero. However, they are not generally implemented for more flexible group comparisons, such as testing if two or more groups of coefficients are equivalent, for example, the summary.glm function in R packages *stats* and Python package *statsmodel* do not support such comparisons. Additionally, most popular GLM tools do not support the rigorous voxelwise hypothesis testing of spatial intensity (or log-transformed intensity), which is a critical focus in CBMA application.

When a GLM involves multiple hypotheses, such as testing estimated group-specific intensity against homogeneity or comparing groups voxel-by-voxel, multiple testing corrections are applied to control for false positives. Without correction, the probability of encountering at least one false positive increases with the number of tests. For example, in localised tests across 228, 483 voxels (within a MNI152 2 mm brain mask), even a 5% false positive rate could result in a substantial number of false positives. In neuroimaging data, multiple testing corrections are applied by controlling either the family-wise error rate (FWER), using the null maximum distribution (Westfall & Young, 1993), or the false discovery rate (FDR) using the Benjamini–Hochberg procedure (Benjamini & Hochberg, 1995). FWER correction is a more stringent approach, as it minimises the chance of any false positives across the entire set of hypothesis tests. However, it often reduces statistical power and leads to fewer significant results, for instance, Bonferroni corrections can be overly conservative and may excessively penalise neuroimaging datasets. In contrast, FDR correction is more powerful in large-scale testing scenarios, where hundreds of thousands of tests are conducted simultaneously, FDR correction improves statistical power and allows for more significant findings while still controlling the overall rate of false discoveries among detected results.

In our previous work on single-group CBMR, both single- and multiple-hypothesis testing of estimated spatial intensity involve the standard error of the estimated intensity or log-transformed estimated intensity. This is derived from the standard error of spatial regression coefficients using the inverse of the Fisher Information matrix, with additional transformations such as the delta method applied. However, in practice, we observed numerical singularity in the Fisher Information matrix, particularly for smaller datasets where the total number of foci is below 200 (Yu et al., 2024). This motivates us to explore parametric bootstrapping as an alternative to parametric inference based on the Fisher Information matrix. By obtaining *p*-values from the tail of the null bootstrap distribution, we avoid the numerical instability caused by extremely small estimated intensity values close to zero. Although this approach increases computational complexity by requiring thousands of bootstrap samples, it provides a more numerical stable solution, See Section 2.1.4 for more details.

### 1.2 Preliminaries

In this section, we provide a brief overview and description of the multi-group CBMR. To simplify notation, we begin with the definition of the single-group CBMR in Section 1.2.1. Following this, we explain how the definition and notation from Section 1.2.1 are extended to the multi-group CBMR setting in Section 1.2.2.

#### 1.2.1 The single-group CBMR

In the simplest single-group settings, a CBMR with M studies (each containing N voxels) is assumed to take the following form:

$$\text{log}\left(\mu_{i}\right)=\text{log}\left[E\left(Y_{i}\right)\right]=X\beta_{i}+\left(Z_{i}\gamma \right){\text{1}}_{N},$$ (1)

where $Y_{ij}$ is the voxel-wise count of foci at voxel j for study i (either 0 or 1 in practice), and *N*−vector $Y_{i}={\left[Y_{i1},Y_{i2},\cdots ,Y_{iN}\right]}^{⊤}$ represents CBMA data for study i. The spatial design matrix X(N×P) is generated with spline parameterisation with P cubic B-spline bases as detailed in Section 1.1.1 (see also Yu et al., 2024), and a study-level covariates matrix Z(M×R) is created with R study-level covariates from M studies followed by standardisation as pre-processing procedure. The estimated intensity is $\mu_{ij}$ for studies i=1,⋯,M and voxels j=1,⋯,N , written as the *N*−vector $\mu_{i}={\left[\mu_{i1},\mu_{i2},\cdots ,\mu_{iN}\right]}^{⊤}$ for study i. This model is identifiable as long as each covariate variable has a mean of zero, allowing X to capture the overall mean. The GLM for all voxels in all M studies is then

$$\text{log}\left[E\left(Y\right)\right]=\left({\text{1}}_{M}⊗X\right)\beta +\left(Z⊗{\text{1}}_{N}\right)\gamma ,$$ (2)

where $Y={\left[Y_{1},Y_{2},\cdots ,Y_{M}\right]}^{⊤}$ is an (*M*×*N*)−vector, containing voxel-wise foci count for all M studies, and ⊗ denotes the Kronecker product. Given that our GLM has millions of rows (MN) and the spatial design matrix has billions of entries (MN×P), we proposed a simplified reformulation of this GLM to reduce complexity and memory requirement. A comprehensive discussion of this reformulation, along with a more detailed introduction to the four stochastic models and the notations used in this section, is provided in our previous work (Yu et al., 2024).

#### 1.2.2 The multi-group CBMR

In the multi-group CBMR setting, a dataset is categorised into multiple groups, we fit group-wise activation intensity functions and generate group-specific statistical maps. Adapting the notation of the previous section, this can be represented as

$$\text{log}\left(\mu_{g\left(i\right)}\right)=\text{log}\left[E\left(Y_{i}\right)\right]=X\beta_{g\left(i\right)}+\left(Z_{i}\gamma \right){\text{1}}_{N},$$ (3)

where the subscript g(i) represents the group that includes study i. In Equation 3, the spatial design matrix, parametrised by spline bases with pre-defined knot spacing, remains fixed across all groups, and the regression coefficient for study-level covariates $\beta_{g(i)}$ is specific to each group. By default, study-level covariates (γ) are modelled as shared among all groups. However, group-specific covariate effects can also be accommodated in two ways:

1. The meta-regression model can be fit separately within each group, yielding independent estimates of γ.
2. Group-specific covariates can be encoded directly in the design matrix by creating separate columns for each group (with entries set to 0’s for studies belonging to other groups).

This formulation allows users to flexibility combine shared and group-specific effects within a single model, depending on the research question. By incorporating group-specific spatial effects while retaining shared global study-level covariates, Equation 3 generalises the conventional form of the single-group CBMR model to the multi-group CBMR framework.

Given a total of M studies divided into G groups, we reorder the study indices according to their respective groups and assume that group g contains $M_{g}$ studies ($M=\sum_{i=1}^{G}M_{g}$). The GLM for all voxels across all $M_{g}$ studies for group g can be represented as

$$\text{log}\left[E\left(Y_{g}\right)\right]=\left({\text{1}}_{M_{g}}⊗X\right)\beta_{g}+\left(Z_{g}⊗{\text{1}}_{N}\right)\gamma ,$$ (4)

where $Y_{g}={\left[Y_{1},Y_{2},\cdots ,Y_{M_{g}}\right]}^{⊤}$ and $Z_{g}={\left[Z_{1},Z_{2},\cdots ,Z_{M_{g}}\right]}^{⊤}$ represent the voxel-wise foci counts and study-level covariates for all $M_{g}$ studies within group *g*. Accordingly, the GLM for all voxels across the M studies is formulated by vertically concatenating Equation 4 for each group. To address the substantial memory and computational demands, a similar reformulation procedure is applied to the multi-group CBMR.

## 2 Methods

This section outlines the computational pipeline employed by CBMR to conduct multi-group meta-regression and meta-inference on CBMA data, as well as the simulations and real-data examples, with results presented in Section 3. To begin, Section 2.1 provides a detailed overview of the stages involved in the CBMR computational pipeline. Next, Section 2.2 describes the simulations designed to evaluate the accuracy and performance of CBMR. Finally, Section 2.3 presents a real-world application using the Cue Reactivity dataset, demonstrating the practical implementation of CBMR.

### 2.1 The CBMR pipeline

**Fig. 1.**
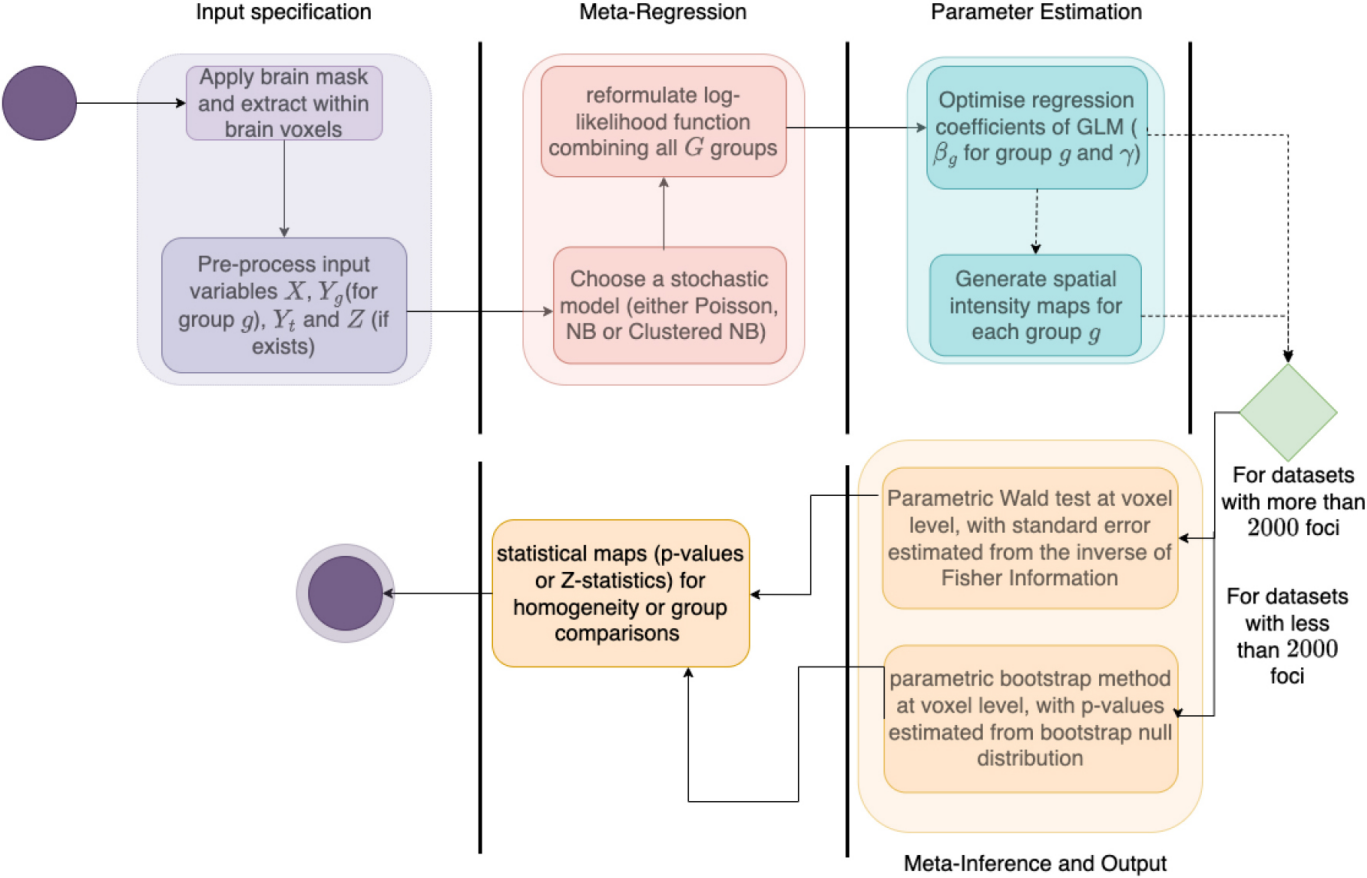
The activity diagram of the CBMR pipeline. The pipeline begins and ends with nodes represented by a dark purple circle and nested dark purple and light purple circles, respectively. Decision nodes are depicted as diamonds, where decisions are made based on whether the number of foci exceeds a specified threshold, while computational stages are represented by vertical bars. Panels separated by these vertical bars correspond to distinct stages within the CBMR pipeline. The entire pipeline is divided into four main stages: input specification, meta-regression, parameter estimation, and meta-inference and output.

Figure 1 presents a visual overview of the CBMR pipeline as an activity diagram. The pipeline is divided into four stages: meta-regression, parameter estimation, and inference and output. Each stage is described in detail in Sections 2.1.1 through 2.1.4. The implementation of CBMR algorithm in the Python package NiMare adheres to these same four stages, as illustrated in Figure 1 (Salo et al., 2022).

#### 2.1.1 Input specification

**Fig. 2.**
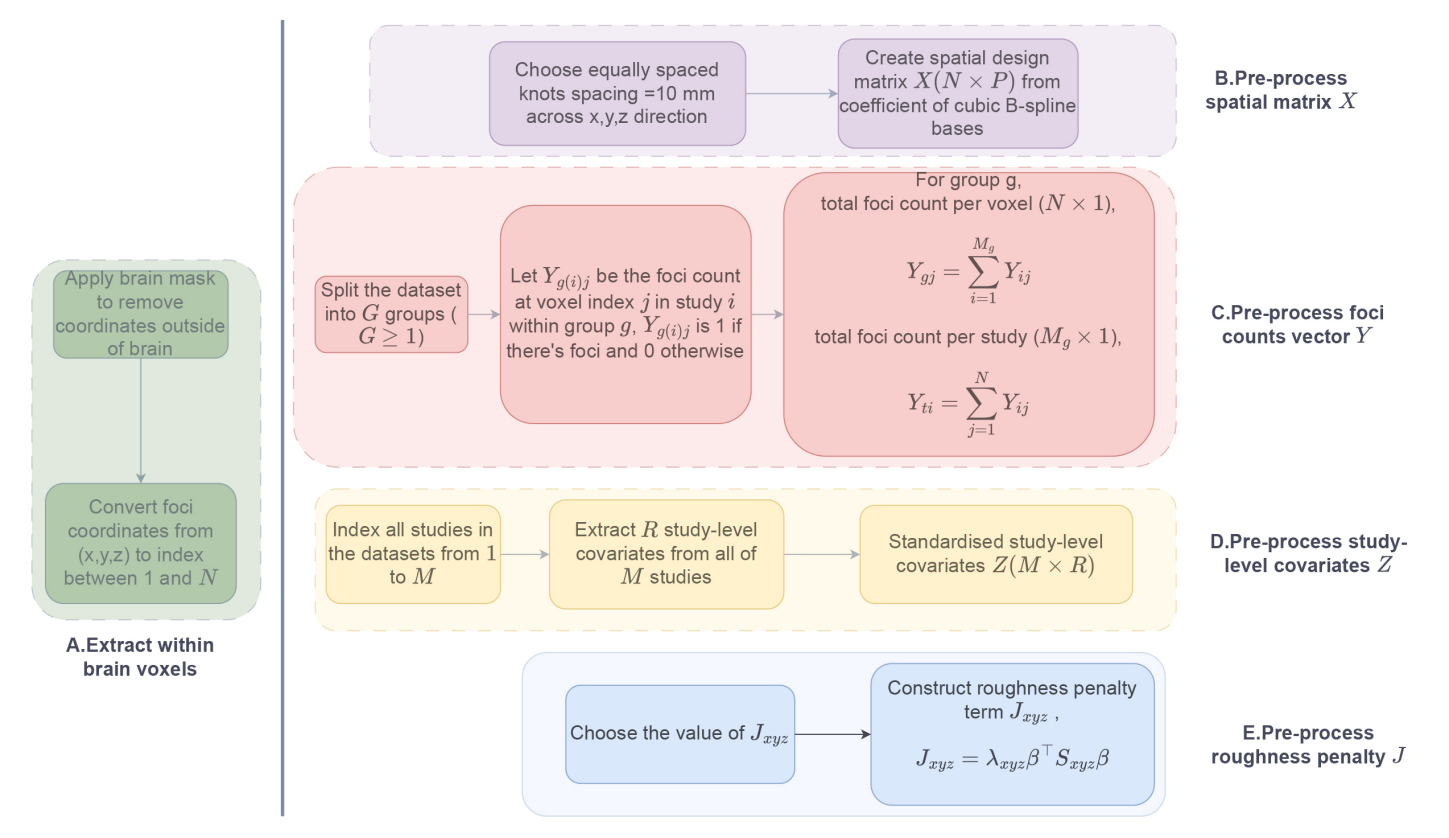
Preprocessing pipeline for multi-group meta-analytic datasets applied before fitting coordinate-based meta-regression (CBMR) framework. Panels A, B, and C are applicable to all datasets and used to generate the spatial design matrix X, total foci count per voxel $Y_{g}\left(N\times 1\right)$ for group g, and total foci count per study $Y_{t}\left(M\times 1\right)$. Panel D is required only when considering the effects of study-level covariates, in which case the covariates matrix Z(M×R) is included. Panel E is recommended for datasets with insufficient foci counts, as it discourages overly complex spatial functions and improves numerical stability.

Figure 2 illustrates the preprocessing steps required to generate all the necessary input variables for the CBMR pipeline. The preprocessing begins by applying a brain mask to exclude all voxels outside the brain. The default brain mask is the MNI152 2 mm template in the code implementation of CBMR. The voxel space has dimensions of 91×109×91 in the x, y, and z directions, resulting in a total of 902, 629 voxels. However, most of these voxels fall outside of the brain mask. Applying the brain mask is, therefore, a crucial step to eliminate redundant voxels and avoid unnecessary computations involving non-brain regions in subsequent processing steps. Next, we select equally spaced knots (with a default spacing of 10mm ) to construct cubic B-spline bases along the x, y, and z directions, assuming the numbers of B-spline bases are $n_{x}$, $n_{y},$ and $n_{z}$, respectively. The coefficients of these basis functions over $v_{x}$, $v_{y},$ and $v_{z}$ voxels yield design matrices with shape $n_{x}\times v_{x}$, $n_{y}\times v_{y}$, and $n_{z}\times v_{z}$ for each dimension. These dimension-specific design matrices are then combined using the tensor product to construct a comprehensive design matrix for further analysis. For more details on the spatial model parametrised by spline bases, refer to Section 1.1.1 and Yu et al. (2024).

In fMRI publications, activation foci are typically reported as their x, y, and z coordinates. A CBMA dataset often contains hundreds or thousands of such foci from numerous studies. It is common and straightforward to compute voxel-wise foci count across the entire brain for each study. Building on the previous single-group CBMR model, our current objective is to investigate group-specific activation intensity functions and perform subsequent CBMR inference analyses. To achieve this, we define multiple groups with clear selection criteria, categorise all studies into these groups, and store voxel-wise foci counts separately for each group to support the analysis. The importance of simplified model factorisation has been highlighted in our previous work, aiming to reduce dimensionality and alleviate computational complexity (Yu et al., 2024). Specifically, we adopt the following three approaches for different stochastic models:

- **Poisson model**: The total voxel-wise foci counts across all studies are assumed to follow a Poisson distribution with the mean equal to the sum of the estimated mean from each study. This leverages the additive property of the Poisson process for computational simplicity and interpretability.
- **NB model**: By matching the first and second moments (mean and variance), we approximate the likelihood function under the assumption that the convoluted voxel-wise foci counts follow a Negative Binomial (NB) distribution.
- **Clustered NB model**: The total log-likelihood function is computed by combining the study-wise log-likelihoods, accounting for intra-study covariance structures introduced by the Clustered NB distribution.

In this work, we adopt the same convention of applying the above model factorisation methods to simplify the log-likelihood functions. However, unlike previous approaches, we first categorise all studies into multiple groups and then apply these factorisation methods at the group level. Following group-level model factorisation, the sufficient statistics are reduced to dimensions no greater than either the number of studies within each group or the number of voxels within the brain mask, as detailed below,

- Let $Y_{gj}=\sum_{i=1}^{M_{g}}Y_{ij}$ be the sum of foci counts at voxel j across all $M_{g}$ studies within the group g, and the *N*−vector *Y_g_* = [*Y_g1_*, *Y_g2_…Y_gN_*]^⊤^;
- Let $Y_{ti}=\sum_{j=1}^{N}Y_{ij}$ be the sum of foci counts for study i across all voxels, and the *M-*vector $Y_{t}=\left[Y_{t1},Y_{t2},\cdots ,Y_{tM}\right]$;
- Let N-vector $\mu_{g}^{X}=\exp(X\beta_{g})$ be the vector of localised spatial effects of studies in group g;
- Let M-vector $\mu^{Z}=\text{exp}\left(Z\gamma \right)$ be the vector of global study-level covariate effects of studies.

In the CBMR pipeline that incorporates the effects of study-level covariates, an additional input variable, $Z_{g}$ (with dimension M×R ), is introduced. This variable is constructed by extracting R study-level covariates from M studies. Common examples of study-level covariates include sample size, year of publication, and participant age. It is important to standardise the study-level covariates to have a mean of 0 and a standard error of 1 during preprocessing. This standardisation ensures that X captures the overall mean, enabling more straightforward and comparable analyses of spatial intensity functions in subsequent steps.

Another potential input variable introduced during CBMR preprocessing is the roughness penalty matrix J (with dimensions P×P ) of spline bases. During optimisation using L-BFGS, we observed challenges with datasets that have insufficient foci counts, where some elements of the group-wise spatial regression coefficients $\beta_{g}$ are driven to highly negative values. This results in an overly flexible and detailed representation of the foci distribution. While such flexibility can model complex patterns, it often leads to overfitting, unnecessarily intricate functions, and causes numerical instability. To address these issues, we incorporate a roughness penalty that penalises overly flexible spatial functions (combinations of spline bases) by discouraging overly complex or “rough” spatial functions. This ensures smoother and more stable solutions. Details on constructing the roughness penalty matrix J are provided in Supplementary Appendix A.1.

#### 2.1.2 Meta-regression

Following the preprocessing of CBMR input variables, the next step is to evaluate different stochastic models to identify the most accurate but parsimonious fit. We will present the statistical formulations, as well as advantages and drawbacks of all the stochastic models proposed in our previous work, except for the Quasi-Poisson model. The exclusion of the Quasi-Poisson model is due to its characteristics as a Quasi-likelihood-based model that requires optimisation using the IRLS algorithm. This approach complicates the implementation of optimisation process, as it does not support the use of L-BFGS algorithm for maximising likelihood functions. Furthermore, as demonstrated in our previous work, the Quasi-Poisson model is similar to the NB model in explainability of excess variation in foci count data but exhibits inferior performance (Yu et al., 2024).

The Poisson model is the simplest stochastic model option in the CBMR pipeline, both in terms of statistical formulation and computational complexity. In practice, the count of foci $Y_{ij}$ (for study i=1,⋯,M and voxel j=1,⋯,N ) is always 0 or 1, which strictly indicates a Binomial model. Therefore, we adopt the Poisson model, inspired by its previous success with Poisson point process and the accuracy of Poisson approximations for low-rate binomial data. One appealing property of the Poisson process is that the sum of multiple Poisson random variables is also Poisson. This allows for practical flexibility: it is equivalent to model either the set of $M_{g}$ study-level counts or the summed counts at each voxel for each group g. Following the model structure outlined in Equation 3, the intensity for voxel j in study i for group g is

$$\begin{array}{l} E[Y_{g(i)j}]=\mu_{g(i)j}^{X}\cdot \mu_{i}^{Z}\\ \log[\mu_{g(i)j}]=\eta_{g(i)j}=X_{j}^{T}\beta_{g(i)}+Z_{i}\gamma , \end{array}$$ (5)

where study i belongs to group g(i) , $Y_{g\left(i\right)j}∼Poisson\left(\mu_{g\left(i\right)j}\right)$, $x_{j}^{⊤}$ is the $j^{th}$ row of spatial design matrix X(N×P), and $\beta_{g(i)}$ is spatial regression coefficients of group g(i) . Under the assumption of independence of counts across studies, the total likelihood function is exactly same if we model the voxel-wise total foci count over studies for each group instead, and the likelihood to be optimised is

$$\begin{array}{l} l(\theta )=l(\beta_{1,}\cdots ,\beta_{G},\gamma )=\sum_{g=1}^{G}l(\beta_{g},\gamma )\\ \;\;\;\;\;\;\;\;\;=\sum_{g=1}^{G}\sum_{j=1}^{N}\left[Y_{gj}\log(\mu_{gj})-\mu_{gj}-\log(Y_{gj}!)\right]\\ \;\;\;\;\;\;\;\;=\sum_{g=1}^{G}Y_{g}^{T}\log(\mu_{g}^{X})+Y_{t}^{T}\log(\mu^{Z})-\sum_{g=1}^{G}\left[1^{T}\mu_{g}^{X}][1^{T}\mu_{g}^{Z}\right]. \end{array}$$ (6)

For more detailed derivations, refer to Supplementary Appendix A.2.1.

While Poisson model is widely used for the regression of count data, foci counts often exhibit over-dispersion in practice, where the variance of the response variable substantially exceeds the mean. In such case, imposing a Poisson model may underestimate the standard error and lead to biased estimates of the regression coefficients. To address this, we propose modelling the count data at each voxel as independently following a group-specific Negative Binomial (NB) distribution, which accounts for excess variance relative the Poisson model (Lawless, 1987). The NB model employs a group-specific single parameter, $\alpha_{g}$, shared across all studies within group g and all voxels, to index the variance in excess of Poisson model. Specifically, for group g, study i, and voxel j, let $\lambda_{g(i)j}$ follow a Gamma distribution with mean $\mu_{g(i)j}$ and variance $\alpha_{g}\mu_{g(i)j}^{2}$. Conditioned on $\lambda_{g(i)j}$ , let $Y_{ij}$ follow a Poisson distribution with mean $\lambda_{g(i)j}$ . Unlike Poisson, the sum of multiple independent NB random variables does not follow an NB distribution. To address this, we propose moment matching approach to approximate the first two moments (mean and variance) of the convolution of NB distributions. This significantly simplifies the log-likelihood function. By matching the first two moments, the approximate NB distribution of the total count of foci across all studies within group g at voxel j is given by $Y_{gj}=\sum_{i=1}^{M_{g}}Y_{ij}\sim NB(r_{gj}^{'},p_{gj}^{'}),$ where

$$r_{gj}^{'}=\frac{\mu_{gj}^{2}}{\alpha_{g}\sum_{i=1}^{M_{g}}\mu_{ij}^{2}},p_{gj}^{'}=\frac{\sum_{i=1}^{M_{g}}\mu_{ij}^{2}}{\alpha_{g}^{-1}\mu_{gj}\sum_{i=1}^{M_{g}}+\sum_{i=1}^{M_{g}}\mu_{ij}^{2}}$$ (7)

with corresponding excess variance for each group g,

$$\alpha_{g}^{'}=\alpha_{g}\frac{\sum_{i=1}^{M_{g}}\mu_{ij}^{2}}{},$$ (8)

which gives rise to the simplified NB log-likelihood function,

$$\begin{array}{l} l(\theta )=l(\beta_{1},\cdots ,\beta_{G},\alpha_{1}^{'},\cdots ,\alpha_{G}^{'},\gamma )=\sum_{g=1}^{G}l(\beta_{g},\alpha_{g}^{'},\gamma )\\ \;\;\;\;\;\;\;\;\;=\sum_{g=1}^{G}\sum_{j=1}^{N}\left[\log\Gamma (Y_{gj}+r_{gj}^{'})-\log\Gamma (T_{gj}+1)-\log\Gamma (r_{gj}^{'}\right)\\ \;\;\;\;\;\;\;\;=+r_{gj}^{'}\log(1-p_{gj}^{'})+Y_{gj}\log(p_{gj}^{'})]. \end{array}$$ (9)

For more detailed derivations, refer to Supplementary Appendix A.2.2.

As a form of “random effects” Poisson model, the NB model incorporates group-specific latent Gamma random variables that introduce independent variation at each voxel. However, in neuroimaging applications, there is often insufficient data to reliably estimate independent voxel-wise variation. We could instead assert that the random (Gamma-distributed) effects are not independent voxel-wise effects, but rather latent characteristics specific to each study within a group. These latent effects represent a shared effect across the entire brain for a given study within the group. This approach is used by a Bayesian CBMA method (Samartsidis et al., 2019) and, in a non-imaging setting, is conceptually similar to a Poisson–Gamma model for two-stage cluster sampling (Geoffroy & Weerakkody, 2001). Unlike the NB model, this method assumes that, at the first stage, each individual study i within group g is associated with a global latent variable $\lambda_{i}$, sampled from a Gamma distribution with mean 1 and variance $\alpha_{g}$. This allows for overdispersion, controlled by the dispersion parameter $\alpha_{g}$ ($\lambda_{i}∼Gamma\left(\alpha_{g}^{-1},\alpha_{g}^{-1}\right)$). At the second stage, conditioned on the global variable $\lambda_{i}$, the observed foci counts $Y_{ij}$ are drawn from a Poisson distribution with mean $\lambda_{i}\mu_{ij}$ ($Y_{ij}|\lambda_{i}∼Poisson\left(\lambda_{i}\mu_{ij}\right)$), where $\mu_{ij}$ represents the expected intensity, parametrised by the spatial regression parameter $\beta_{g}$ and covariates regression coefficients γ. The covariance structure between foci within a study is captured by this two-stage hierarchical Clustered NB model. It is determined by both the expected intensity of the foci locations and the group-specific dispersion parameter $\alpha_{g}$. Specifically, the covariance for study i and $i^{\prime }$ within group g, and for distinct voxel j and $j^{\prime }$ , is given by

$$\{\begin{array}{c} \text{C}\left(Y_{ij},Y_{i^{\prime }j^{\prime }}\right)=\alpha_{g}\mu_{ij}\mu_{i^{\prime }j^{\prime }},\;if\;\;i=i^{\prime }\\ \text{C}\left(Y_{ij},Y_{i^{\prime }j^{\prime }}\right)=0,\;if\;\;i\neq i^{\prime }. \end{array}$$ (10)

The reformulated log-likelihood is the sum of log-likelihood over independent studies,

$$\begin{array}{l} I(\theta )=1(\beta_{1},\cdots ,\beta_{G,}\alpha_{1}\cdots ,\alpha_{G}\gamma )=\sum_{g=1}^{G}I(\beta_{g},\alpha_{g},\gamma )\\ \;\;\;\;\;\;\;\;\;\;=\sum_{g=1}^{G}[M_{g}\alpha_{g}^{-1}\log(\alpha_{g}^{-1})-M_{g}\log\Gamma (\alpha_{g}^{-1})+\sum_{i=1}^{M_{g}}\log\Gamma (Y_{ti}+\alpha_{g}^{-1})\\ \;\;\;\;\;\;\;\;\;=\sum_{i=1}^{M_{g}}\left(Y_{ti}+\alpha_{g}^{-1})\log(\mu_{ti}+\alpha_{g}^{-1})+Y_{g}^{T}\log(\mu^{X})]+Y_{t}^{T}\log(\mu^{Z}\right). \end{array}$$ (11)

Despite its motivation to induce intra-study dependence, the Clustered NB model relies on the strong assumption that excess variance is fully captured by the global dispersion parameter $\lambda_{i}$. However, it cannot accommodate voxel-wise independent excess variance within a study.

#### 2.1.3 Parameter estimation

The most computationally intensive stage of the CBMR pipeline is the estimation of the unknown model parameters $\left(\beta_{g},\alpha_{g},\gamma \right)$ for each group g. A common approach for estimating these group-specific parameters is Maximum Likelihood Estimation (MLE), based on reformulated log-likelihood functions tailored to each stochastic model described in Section 2.1.2. To efficiently optimise these parameters, the CBMR pipeline employs the L-BFGS algorithm, a quasi-Newton method well suited for problems involving large-scale datasets and high-dimensional parameter spaces. By approximating the Hessian matrix rather than computing and storing it directly, the L-BFGS algorithm achieves significant computational efficiency, making it ideal for the CBMR pipeline (Liu & Nocedal, 1989). Considering the log-likelihood function is non-convex for both the NB model and the Clustered NB model, a more cautious optimisation strategy is adopted. Specifically, a smaller learning rate is used during L-BFGS optimisation to reduce the risk of the algorithm becoming trapped in a local optimum rather than converging to the global optimum.

For the Poisson model in CBMR, the group-specific spatial regression coefficient $\beta_{g}$ is initialised either with random values uniformly distributed within the range [−0.01, 0.01] or with values assuming spatial homogeneity of foci locations. Both initialisation strategies allow the L-BFGS algorithm to converge effectively. To address the non-convexity of log-likelihood functions for NB and Clustered NB models, we propose using the optimised spatial regression coefficient $\beta_{g}$ from the Poisson model as the initialisation for these two models, improving the stability and robustness of the optimisation process. During optimisation, we iteratively optimise the group-wise dispersion parameter $\alpha_{g}$ while keeping the group-specific spatial regression coefficient $\beta_{g},$ and, if applicable, the coefficient of study-level covariates γ fixed. Subsequently, $\alpha_{g}$ is fixed, and other variables are optimised in alternating iterations until convergence. Pseudocode for these alternating iterations is provided by 1.

**Algorithm 1.** Alternating iterations for CBMR with NB model or Clustered NB model.

Assign initial estimates to group-specific parameters $\beta_{g}$, $\alpha_{g},$ and group-shared γ **while** *Current* l(θ) *and previous* $l_{prev}\left(\theta \right)$ *differ by more than a predefined tolerance* **do** Evaluate the previous log-likelihood using $l_{prev}\left(\theta \right)=\sum_{g=1}^{G}l_{g}\left(\beta_{g},\alpha_{g},\gamma \right)$ **while** *Current* $l\left(\alpha_{g}\right)$ *and previous* $l_{prev}\left(\alpha_{g}\right)$ *differ by more than predefined tolerance ($1e^{-9}$ by default)* **do** Update the group-wise dispersion parameter $\alpha_{g}$ for each group g using L-BFGS algorithm, while keeping $\beta_{g}$ and γ (if applicable) fixed. **end** **while** *Current* $l\left(\beta_{g},\gamma \right)$ *and previous* $l_{prev}\left(\beta_{g},\gamma \right)$ *differ by more than predefined tolerance ($1e^{-9}$ by default)* **do** Update the group-specific parameters $\beta_{g}$ and the group-shared parameter γ (if applicable), while keeping $\alpha_{g}$ fixed. **end** Recompute the current log-likelihood values l(θ) and calculate the difference from the previous log-likelihood values. **End**

In the implementation of the CBMR parameter estimation stage, we use the built-in function *scipy.optimize. minimize(method=‘L-BFGS-B’)* from Scipy to minimise the objective function (negative log-likelihood function). This L-BFGS function was chosen for its well-documented and user-friendly interface, which simplifies integration into the pipeline. To address the increased computational demands of the parametric bootstrap method (see Section 2.1.4 for details), we also implemented parallelisation to accelerate computation. Furthermore, we implemented the code in JAX to take advantage of its automatic differentiation capabilities. This allows for efficient approximation of the observed Fisher information matrix using the optimised regression coefficients for inference based on the Wald test (see Section 2.1.4 for details), without the need to explicitly derive the Hessian matrix of the log-likelihood function.

Using the optimised CBMR regression coefficients, we can construct group-specific estimated intensity maps to intuitively visualise the brain activation patterns. For more rigorous inference, allowing the identification of brain regions with significant *p*-values from statistical maps, we will further implement meta-inference pipelines, with further details provided in Section 2.1.4.

#### 2.1.4 Meta-inference and output

The final stage of the CBMR pipeline involves performing inference (for both the homogeneity test and group comparison test) on the group-specific estimated intensity maps and outputting the analysis results as statistical maps in NIfTI format. To conduct voxel-wise hypothesis testing for both types of test, CBMR adopts an approach similar to that used in the popular GLM python package *statsmodels* and the R function *glm()*. In this approach, the group-specific estimated spatial intensity $\mu_{g}^{X}$ or its log-transformed counterpart $\eta_{g}^{X}$ are used to construct test statistics at voxel-wise level, as well as their standard errors.

Assuming a contrast matrix C(m×S) is provided for S involved groups, a voxel-wise null hypothesis $H_{0}\;:C{\hat{\theta }}_{j}=0_{m\times 1}$ for voxel j can be specified. For simplicity, we assume that any redundant columns containing only zero elements (corresponding to groups not involved in the contrast) are removed before proceeding with the analysis. CBMR computes the corresponding test statistics as

$${\left(C{\hat{\theta }}_{j}\right)}^{⊤}{\left(CV_{j}C^{⊤}\right)}^{-1}\left(C{\hat{\theta }}_{j}\right)\overset{D}{\to }\chi_{m}^{2}$$ (12)

where ${\hat{\theta }}_{j}$ represents either the estimated intensity ${\left[{\hat{\mu }}_{1j}^{X},\cdots ,{\hat{\mu }}_{Sj}^{X}\right]}^{⊤}$ or its log-transformed value ${\left[{\hat{\eta }}_{1j}^{X},\cdots ,{\hat{\eta }}_{Sj}^{X}\right]}^{⊤}$, in practice, we recommend using the log-transformed values ${\hat{\eta }}_{gj}^{X}$, as they correspond to the linear response of GLMs, avoiding the additional approximation required to transform ${\hat{\eta }}_{gj}^{X}$ to ${\hat{\mu }}_{gj}^{X}$ for estimating standard errors. The inverse of the Fisher information gives the asymptotic variance of the estimates of spatial regression coefficients $\beta_{1},\cdots ,\beta_{S}$ for the S involved groups. Leveraging the deterministic structure of GLMs ${\hat{\eta }}_{g}^{X}=X\beta_{g}$, we approximate the variance of ${\hat{\eta }}_{g}^{X}$ for group g as $X^{⊤}Var\left(\beta_{g}\right)X$ , where X is the spatial design matrix. Additionally, $V_{j}\left(S\times S\right)$ represents the covariance matrix constructed from the estimated variance of ${\hat{\eta }}_{g}^{X}$ at the $j^{th}$ voxel across all S groups. The degrees of freedom for the statistical test are determined by the number of rows in the contrast matrix C. To calculate the corresponding *p*-values, the test statistics are approximated using a chi-square distribution.

In scenarios where only one group is involved (m=S=1 ), the statistical test simplifies to a Wald test, with the null hypothesis formulated as $C\left({\hat{\theta }}_{j}-\theta_{0}\right)=0$ . This can be further simplified to the following form:

$$W_{j}=\frac{{\hat{\theta }}_{j}-\theta_{0}}{\text{SE}\left({\hat{\theta }}_{j}\right)},$$ (13)

where ${\hat{\theta }}_{j}$ represents either the estimated intensity ${\hat{\mu }}_{gj}^{X}$ for the involved group g or its log-transformed value ${\hat{\eta }}_{gj}^{X}$. The corresponding voxel-wise *p*-value $p_{j}$ is calculated under the assumption that the Wald test statistics $W_{j}$ follows a standard normal distribution.

Despite efforts to improve numerical stability during the optimisation process, such as adding a roughness penalty to prevent coefficients from being driven to highly negative values, we observed that approximating group-wise spatial regression coefficient by inverting Fisher Information matrix often results in numerical instability. This is particularly prevalent in datasets with an insufficient number of foci, with a practical threshold of at least 200 foci required for reliable inference. The instability arises due to the high dimensionality of the Fisher Information matrix, which can have hundreds or even thousands of elements corresponding to the spline basis functions. For datasets with a low foci count, the Fisher Information matrix can become numerically singular because most voxels have near-zero intensity estimates. We have experimented with several approaches to improve this instability, including adding a small epsilon ($10^{-6}$ ) or 1% of the largest diagonal element to the diagonal of the Fisher Information matrix, and computing the Fisher Information under the assumption that the null hypothesis of homogeneity is True. However, these methods consistently resulted in underestimation of the variance of voxel-wise spatial intensity, resulting in invalid *p*-values.

Given these challenges, we are now exploring parametric bootstrap methods as an alternative for meta-inference, rather than relying on statistical tests based on the inverse of the Fisher Information matrix. The parametric bootstrap is a resampling-based statistical technique that estimates the sampling distribution of a statistic without requiring strong parametric assumptions about the underlying data distribution. Specifically, for group-wise homogeneity test, the bootstrap process involves the following steps: for each bootstrap sample, foci are randomised under the assumption of spatial homogeneity, following a Binomial process. The CBMR regression is refitted to obtain group-wise estimated intensity values or their log-transformed values at voxel level. This procedure is repeated at least 1,000 times to generate the bootstrap null distribution. Under the null distribution $H_{0}\;:\eta_{gj}^{X}=\eta_{g0}$ or $\mu_{gj}^{X}=\mu_{g0}$ , the observed values of $\eta_{gj}^{X}$ or $\mu_{gj}^{X}$ for group g are compared with the bootstrap null distribution. The *p*-values are then calculated as the probability of observed test results as extreme as the actual results, assuming the null hypothesis is true. While for group comparison tests, a similar bootstrap procedure is applied with a slight modification: under the null hypothesis $\eta_{Aj}=\eta_{Bj}$ between group A and B, we first combine all foci counts from both groups to estimate a shared activation intensity function. Data are then regenerated from the chosen stochastic model associated with the CBMR regression, ensuring the total number of studies remains the same as before for both groups. The model is refitted for each bootstrap sample. Repeating this procedure generates the bootstrap null distribution, and *p*-values are calculated by comparing the actual results with the bootstrap null distribution. We assert that this method avoids the numerical issues encountered during the inference stage, although at the cost of increased computational requirements. Its validity and effectiveness are demonstrated in Sections 2.2.

Nevertheless, the precision of the parametric bootstrap method is fundamentally constrained by the number of resamples, B, as the smallest attainable *p*-value is 1/​B . To address this limitation, we adopt a tail-fitting procedure based on the Generalised Pareto Distribution (GPD). The GPD arises naturally from Extreme Value Theory (EVT), which establishes that the distribution of exceedances over a sufficiently high threshold converges to a GPD, regardless of the underlying data-generating process. By fitting the GPD to the upper tail of the empirical bootstrap null distribution beyond a pre-specified threshold, we can extrapolate the distribution’s behaviour more accurately. This allows stable estimation of very small *p*-values (p<1/​B ), thereby extending the effective resolution of the bootstrap procedure.

Additionally, we are also interested in investigating the global effects of study-level covariates on group-wise spatial activation functions. For example, we aim to assess whether there is a global effect of the (square root of) sample size on spatial activation functions, or whether the influence of (square root of) sample size is stronger than that of publication year. To address these questions, we perform hypothesis testing on one or more elements of the regression coefficient vector γ, which captures the effects of the study-level covariates. Similarly to the voxel-wise hypothesis testing of spatial intensity in Equation 12, this is achieved using a contrast matrix $C_{\gamma }\left(m\times s\right)$, where s denotes the number of relevant study-level covariates after excluding irrelevant ones. The contrast matrix $C_{\gamma }$ allows for the specification of flexible hypotheses. Under the null hypothesis $H_{0}:C_{\gamma }\gamma =0_{m\times 1}$ , the test statistic is given by

$${\left(C_{\gamma }\hat{\gamma }\right)}^{⊤}{\left(C_{\gamma }Cov\left(\hat{\gamma }\right)C_{\gamma }^{⊤}\right)}^{-1}\left(C_{\gamma }\hat{\gamma }\right)\overset{D}{\to }\chi_{m}^{2},$$ (14)

where $Cov(\hat{\gamma })$ represents the covariance structure of elements in $\hat{\gamma }$, and the *p*-values can be approximated using a chi-square distribution with m degrees of freedom. Note that the issue of inverting a numerically singular Fisher Information matrix is unlikely to arise when performing inference on the regression coefficients of study-level covariates. This is because γ typically contains only a few elements (fewer than 5), resulting in a Fisher Information matrix of low dimensionality. Furthermore, since most of elements in γ are unlikely to be simultaneously close to 0, ensuring the Fisher Information matrix is not numerically singular. Therefore, we believe it is unnecessary to use bootstrap methods for inference on study-level covariates.

### 2.2 Simulation methods

In order to quantitively evaluate and demonstrate the computational accuracy and efficiency of CBMR, extensive simulations were conducted across 12 settings. Simulated data were generated for three spatial configurations: a 2-dimensional grid consisting of 100×100 voxels, a 3-dimensional grid consisting of 100×100×100 voxels, a 3-dimensional grid within an MNI152 2 mm brain mask, containing 228, 483 voxels. Each configuration was analysed under four data generation designs. These data generation designs combined two key factors: the underlying intensity function and the spatial patterns for data generation. The underlying intensity function for generating CBMR data is either high (an average total foci count of approximately 1, 000 per study) or low (an average total foci count of approximately 10 ). Data generation followed either a homogeneous spatial intensity assumption or a scenario with two Gaussian bump signals overlaid on a background constant intensity function. The high-intensity and spatial homogeneity setting is primarily used as a sanity check, in contrast, the low-intensity and two bump signals setting is designed to evaluate model performance under more realistic conditions that closely reflect real-world datasets. For each simulation setting, the simulated data include 3 groups with identical underlying intensity functions, but different numbers of studies: 100 , 100, and 500 , respectively. Following data generation, CBMR regression is performed using either the Poisson or NB model, as the Clustered NB model has been shown to be incapable of accommodating voxel-wise independent excess variance within a study. Statistical tests are conducted using either standard error estimates derived from the inverse of the Fisher Information matrix or a bootstrap approach with 1, 000 bootstrap samples.

In each simulation setting, the spatial design matrix X (P=2,624 ) is constructed using cubic B-spline bases with knot spacing of 10 mm. This design matrix is fixed and applied consistently across all groups in every simulation setting. The effect of study-level covariates is assumed to exist in all settings, with their values are generated uniformly within the range [−1, 1] and standardised to have a mean of 0 and a standard deviation of 1, allowing X to capture the overall mean. During the optimisation process in each simulation, the group-specific spatial regression $\beta_{g}$ and the shared regression coefficient γ across all groups are estimated, and then used to construct the group-specific intensity maps, as defined by Equation 4.

In order to evaluate the accuracy and performance of parameter estimation in each simulation setting, we conduct meta-inference for both homogeneity test within each group and the group comparison test between any two groups. These tests were performed at the voxel level using two inference approaches: (i) parametric statistical tests, as detailed in Equation 12, and (ii) the parametric bootstrap method. After obtaining voxel-wise *p*-values, they were sorted in an ascending order and visualised using a PP-plot to compare the probability distribution of the observed and theoretical *p*-values. The x-axis represents the theoretical distribution (a uniform distribution between 0 and 1), while the y-axis represents the observed distribution. If the two distributions are similar, the sorted pairs of observed and theoretical *p*-values are expected to align closely along the 45-degree diagonal line (y=x ). Deviations from this diagonal indicate discrepancies between the observed data and the theoretical distribution. Additionally, the group-specific estimated intensity maps produced by CBMR were compared using the mean absolute difference across the whole brain image to assess the accuracy of the CBMR regression stage. Finally, the computational time for the two inference approaches was recorded for comparison.

In summary, the simulations we have described evaluate CBMR in three key aspects: (i) the accuracy of parameter estimation during the regression stage, (ii) the performance of parametric statistical tests and parametric bootstrap method under various data-generation settings, and (iii) computational time required to run the CBMR pipeline. All reported results were obtained using an HPC cluster with Intel(R) Xeon(R) Gold 6126 2.60HZ processors each with 16 GB RAM.

### 2.3 Real data methods

As a demonstration of the large-scale capabilities of CBMR, here we present an example involving a more complex data than those considered in the simulation discussed in Section 2.2. In this example, we utilise a meta-analytic cue-reactivity dataset, as the cue-reactivity paradigm is a widely used neuroimaging probe that elicits brain activity associated with attentional, affective, and reward processes in response to appetitive stimuli. Literature search for visual cue-reactivity fMRI studies focused on drugs of abuse or natural rewards published up to August 2020. Cue types include nicotine, alcohol, cannabis, cocaine, heroin, food, or sexual stimuli. This dataset includes 546 contrasts examining drug-neutral (“drug”, n=163 ), natural-neutral (“natural”, n=110 ) and reward-neutral (“reward”, n=273 ). Relevant study-level information was recorded, including participant age, sex, cue type, MRI scanner field strength, and processing software (Hill-Bowen et al., 2021).

Here, we address two primary research questions using either voxel-level group-wise spatial homogeneity tests or group comparison tests between multiple groups:

- Where are the regions of activation associated with a specific group of cue-reactivity stimuli (e.g., drug-related stimuli) that show stronger estimated intensity than average, under the assumption of spatial homogeneity?
- Where do differences exist in activation regions between two stimulus types within the cue-reactivity dataset (e.g., differences between drug and natural stimulus groups)?

At the pre-processing stage, all 546 contrasts are categorised into three groups based on their respective visual stimulus types. Foci located outside of the MNI152 2 mm brain mask are removed, and a spatial design matrix X(P=2,624) is constructed using cubic B-splines with a knot spacing of 10 mm. In this experiment, we include the square root of the sample size and publication year as study-level covariates. These covariates are standardised to have a mean of 0 and a standard deviation of 1 before being integrated into the CBMR pipeline. At the CBMR regression stage, either Poisson or Negative Binomial (NB) model is employed for parameter estimation. This involves optimising the group-specific regression coefficients $\beta_{g}$ for each group g, the group-shared regression coefficients for the effects of the study-level covariates, and if the NB model is employed, the group-specific overdispersion parameter $\alpha_{g}$. Using these estimates, group-specific intensity maps are then constructed for each group according to Equation 4. At the CBMR inference stage, both group-wise homogeneity tests and group comparison tests between any two groups are performed. Voxel-wise *p*-values are obtained using either statistical tests, as described in Equation 12, or by comparing the observed data to null distributions generated via parametric bootstrap methods with 1, 000 bootstrap samples. Activation maps (for significant uncorrected *p*-values under the 5% significance level) generated by these two inference methods are compared against those generated by ALE. For the group-wise homogeneity test, ALE activation maps are computed using the default full-width half maximum (FWHM) settings based on sample size, as described in Eickhoff et al. (2012). For group comparison, ALE subtraction analysis is employed. Both methods are implemented using the built-in functions of the Python package NiMARE (Salo et al., 2022). Additionally, we investigate the global effects of sample size and publication year on the group-wise intensity functions, analysing if these effects are significant, as well as comparing the strength for each group g.

The primary goal of the analyses described above is to demonstrate the practical application of CBMR and to highlight its efficiency and scalability through a real-world example. To evaluate computational efficiency, the time required for parameter estimation during the regression stage was recorded for both inference methods: parametric statistical tests and parametric bootstrap. In Section 3.2, results are reported for Likelihood Ratio tests, along with alternative model selection criteria such as AIC and BIC, which takes the model complexity into consideration. All analyses were conducted on an HPC cluster with Intel(R) Xeon(R) Gold 6126 2.60HZ processors each with 16 GB RAM.

## 3 Results

### 3.1 Simulation results

#### 3.1.1 Parameter optimisation

Across the 12 simulation settings outlined in Section 2.2 (three spatial configurations combined with 4 data generation schemes), all parameter estimates produced by CBMR regression closely matched the ground truth. For consistency and clarity, we focus on showcasing results from the experimental design involving CBMR regression with the NB model applied to a three-dimensional brain image, as all designs demonstrated similar patterns. The observed absolute bias for intensity function estimation, averaged across all 1, 000 bootstrap samples and voxel locations, is presented in Table 2. We noticed that settings with low underlying intensity and intensity functions with two bump signals posed greater challenges for CBMR regression, as reflected in larger absolute bias values. However, all results remain within the magnitude of $10^{-4}$ , demonstrating the validity and accuracy of CBMR regression for multiple groups under various experimental conditions.

**Table 2.** Bias for CBMR intensity function estimation under different conditions: High or low underlying intensity levels, combined with either spatially homogeneous intensity functions or intensity functions with two bump signals.

	Spatial homogeneous intensity	With two bump signals
High intensity	$1.1501\times 10^{-4}$	$2.4335\times 10^{-4}$
Low intensity	$1.6793\times 10^{-4}$	$6.4429\times 10^{-4}$

#### 3.1.2 Computation time

We emphasise that, after model re-factorisation, neither the number of foci nor the number of studies affects the computation time. This is because the sufficient statistics are reduced to the group-wise vector of voxel-wise total foci counts across all studies within group g ($y_{g}$, N×1 ) and the vector of total foci counts across all voxel locations within a study ($y_{t}$, M×1 ). Only the number of groups influences the computation of log-likelihood function during each iteration. Therefore, we assert that our CBMR regression stages scale efficiently with the number of studies or foci. Moreover, a larger number of studies or foci improve numerical stability and accelerate convergence during the optimisation process.

As a computationally efficient alternative to Bayesian model-based meta-regression methods, one of the key advantages of our CBMR pipeline is its simple, intuitive statistical structure and scalability. Our experiments demonstrate that the optimisation in meta-regression with multiple groups takes approximately 30 minutes on an NVIDIA GTX 1080 Graphics Card—a significant improvement compared with some Bayesian model-based methods, which require roughly 30 hours on an NIVDIA Tesla K20c GPU card (Samartsidis et al., 2019). However, in experimental settings with an insufficient number of foci (e.g., low underlying intensity functions with either spatial homogeneity or two bump signals in our setting), we consider using parametric bootstrap methods as an alternative. This is due to the occurrence of numerical singularities in the Fisher Information matrix, which prevents the subsequent meta-inference stage. Nonetheless, the parametric bootstrap method is computationally intensive, as it requires repeated data simulations (e.g., 1, 000 bootstrap samples in our experiment) and model refitting for each sample to obtain the bootstrap null distribution. In practice, we implemented parallelisation on HPC clusters to accelerate model re-fitting. Running model refitting on five bootstrap samples in parallel on a single HPC cluster node with Intel(R) Xeon(R) Gold 6126 2.60HZ processors takes approximately 40 minutes. Experiments based on parametric bootstrap methods are feasible only with parallelisation and the availability of hundreds of HPC cluster nodes. However, this approach is as computationally intensive as or even more than the Bayesian model-based methods and is not easily accessible to users without HPC cluster resources. As a result, the CBMR regression loses one of its key advantages—computational efficiency, when applied to small meta-analytic datasets with less than 200 foci per group.

#### 3.1.3 Validation of the meta-inference stage

Following the simulation settings described in Section 2.2, we validate the accuracy of the meta-inference pipeline by evaluating PP-plots of voxel-wise *p*-values under each simulation scenario. These *p*-values are computed either using parametric statistical tests described in Equation 12 or through the parametric bootstrap method. A perfect alignment with the y=x line would indicate that the meta-inference stage produces valid outcomes, thereby providing confidence to apply the same inference procedure to real datasets.

**Fig. 3.**
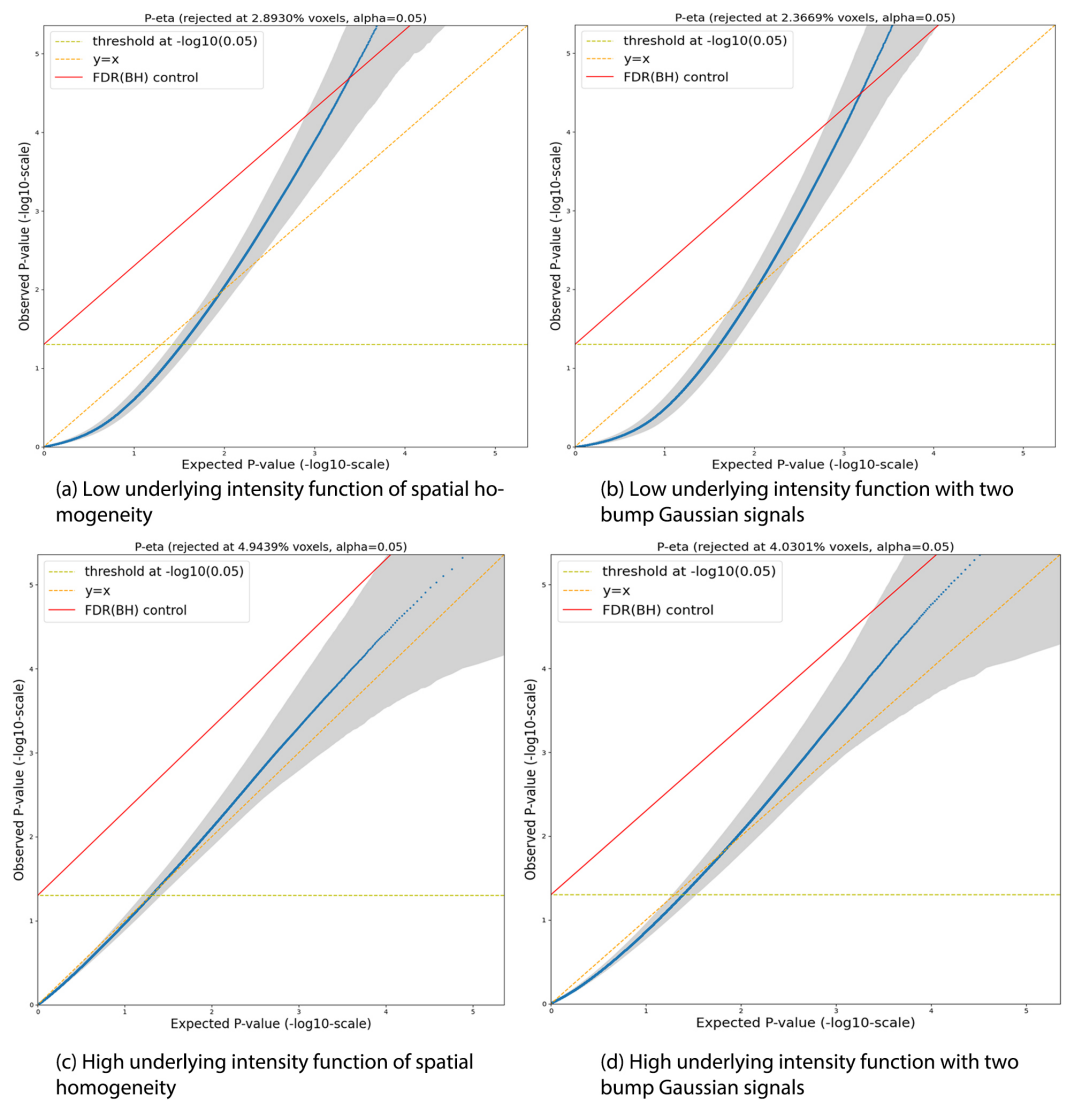
PP-plots illustrating group comparison under two underlying intensity function settings: low (1,000 vs. 5,000 foci) or high (100,000 vs. 500,000 ). Each setting includes both spatially homogeneous and two-bump signal configurations. The plots are based on *p*-values obtained from statistical tests described in Equation 12.

Since the PP-plots are very similar across the 12 scenarios, we only present results for a representative setting: CBMR inference using the parametric statistical test described in Equation 12. This setting compares estimated intensity functions between two groups with identical underlying intensity functions. Foci locations are simulated at different overall intensities (1, 000 vs. 5, 000 foci, or 100, 000 vs. 500, 000 foci) and exhibit either spatial homogeneity or two Gaussian bump signals. Figure 3 displays four $-{\text{log}}_{10}$ PP-plots corresponding to different underlying intensity functions in this simulation setting. The plots include the y=x line (dashed diagonal line), the 5% significance (dashed horizontal line) and the FDR 5% boundary (solid diagonal line); and gray-shaded areas indicating the point-wise 95% prediction intervals. The results show that for scenarios with low underlying intensity functions (both spatial homogeneous and with two bump signals), *p*-values $>0.05\approx 10^{-1.3}$ can skew conservative, while extreme *p*-values can skew liberal. This poor behaviour is observed in both spatially homogeneous and bump signal cases, particularly when the number of foci in insufficient. Conversely, for scenarios with high underlying intensity functions, the PP-plot lines are only slightly skewed, and the y=x line falls within the point-wise 95% prediction intervals. These results support the observation that PP-plots exhibit poor behaviour when the number of foci falls below a certain threshold (1, 000 in our previous one-group CBMR experiment; Yu et al., 2024). In such cases, inference results based on parametric statistical tests become unreliable due to numerical singularity in the Fisher information matrix encountered during practical implementation.

**Fig. 4.**
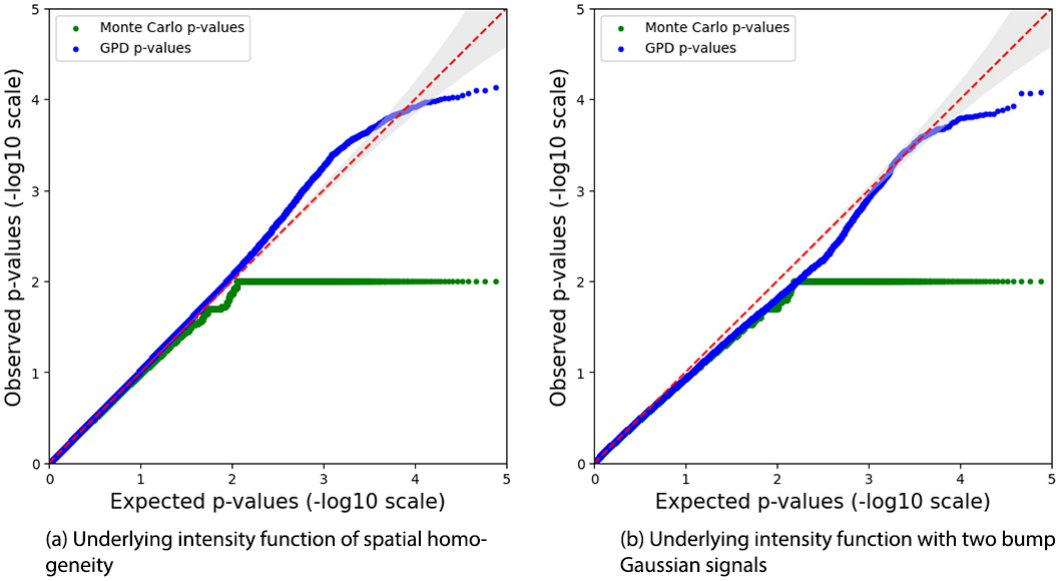
PP-plots illustrate group comparisons (1,000 vs. 5,000 foci) under two types of underlying low-intensity functions: spatially homogeneous and with two Gaussian bump signals. The plots are generated from *p*-values obtained via a parametric bootstrap procedure. The green line corresponds to the bootstrap method without tail fitting, where precision is limited by the number of resamples. The blue line shows the fitted tail using a Generalised Pareto Distribution (GPD). The red line indicates the line of identity (y=x ).

For datasets with an insufficient number of studies or foci, we recommend using the parametric bootstrap method instead, thereby avoiding inverting the Fisher Information matrix (See Section 2.1.4 for details). Figure 4 presents the results from the same representative simulation setting as above, focusing on the more challenging scenario with a low underlying intensity function (with an average of 10 foci sampled per study). The PP-plots for both foci patterns (spatially homogeneous and two bump signals) align closely with the line of identity y=x , with extreme *p*-values exhibiting only a slight conservative skew. The PP-plot generated by the standard bootstrap approach (green line) flattens after $-{\text{log}}_{10}\left(B\right)$, reflecting the limited precision of 1/​B , where B is the number of bootstrap resamples. However, fitting the tail with a Generalized Pareto Distribution (GPD) (blue line) significantly improves precision. These results indicate that the parametric bootstrap method provides an effective alternative to parametric statistical tests for small datasets, although at the cost of increased computational time, with precision further improved through GPD-based tail-fitting approximation.

### 3.2 Real data results

#### 3.2.1 Model comparison

We evaluate the goodness of fit among two likelihood-based stochastic models (Poisson and NB model) by comparing their maximised log-likelihood values. Our analysis shows that CBMR using the NB model outperforms Poisson model on Cue Reactivity dataset, when comparing the maximised total log-likelihood values across multiple groups. This is not surprising, as the NB model accounts for the anticipated excess variance relative to the Poisson model at voxel level. Given the nested relationship between the Poisson and NB models (with the group-specific dispersion parameter $\alpha_{g}=0$ for group g in the NB model), we also performed a Likelihood Ratio Test (LRT) to evaluate the trade-off between model sufficiency and complexity. The LRT results indicate that the null hypothesis—the simpler nested model (Poisson) is as good as the full model (NB)—is strongly rejected for the Cue Reactivity dataset, with *p*-values less than $10^{-8}$ .

#### 3.2.2 Analysis results

**Fig. 5.**
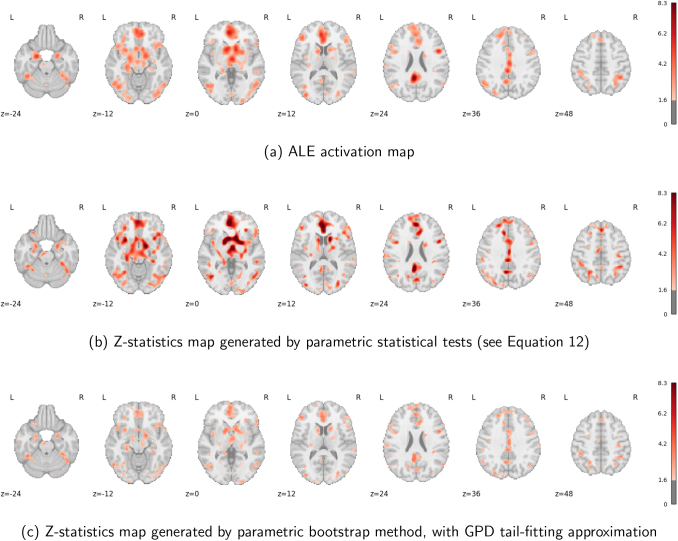
Comparison of activation regions for the **Drug-Neutral group**: ALE activation map, parametric statistical tests, and parametric bootstrap method (with GPD tail-fitting). The activation maps are presented in Z-scores, showing regions with uncorrected *p*-values under 5% significance level.

**Fig. 6.**
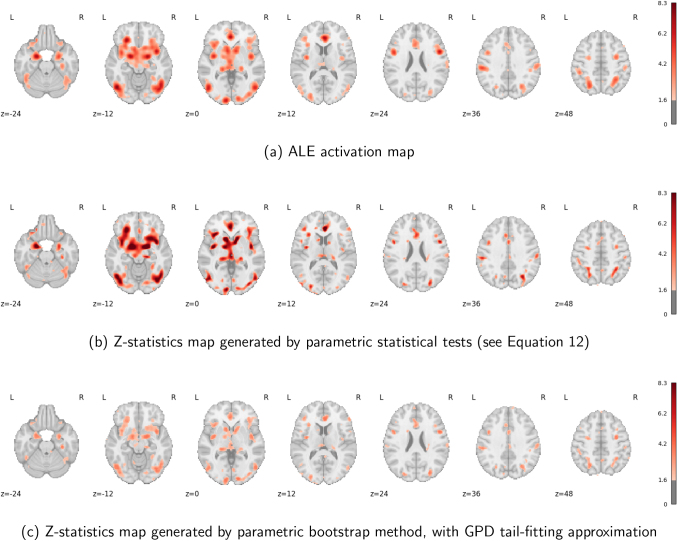
Comparison of activation regions for the **Natural-Neutral group**: ALE activation map, parametric statistical tests, and parametric bootstrap method (with GPD tail-fitting). The activation maps are presented in Z-scores, showing regions with uncorrected *p*-values under 5% significance level.

**Fig. 7.**
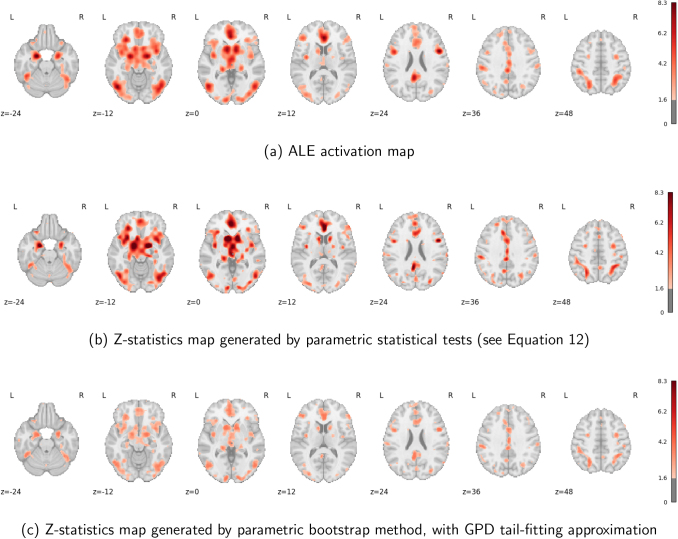
Comparison of activation regions for the **Reward-Neutral group**: ALE activation map, parametric statistical tests, and parametric bootstrap method (with GPD tail-fitting). The activation maps are presented in Z-scores, showing regions with uncorrected *p*-values under 5% significance level.

**Fig. 8.**
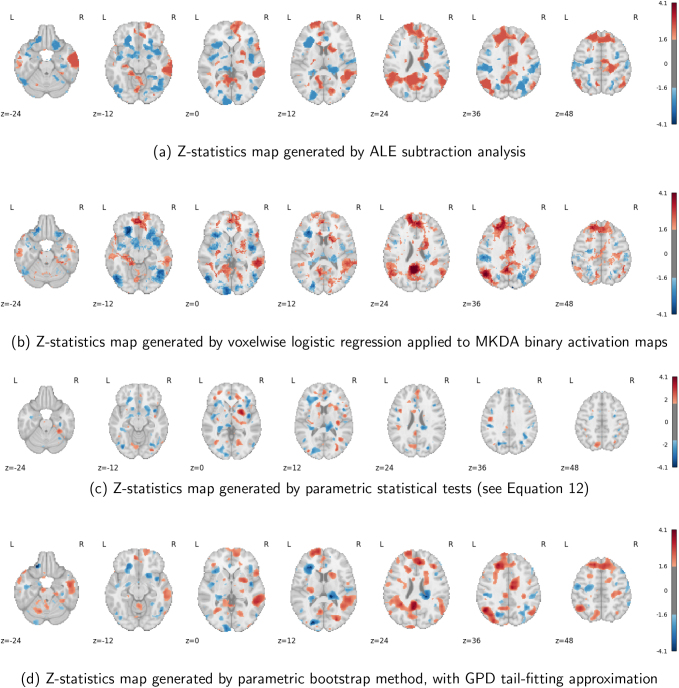
Difference in activation regions between the **Drug-Neutral** and **Natural-Neutral** groups: ALE subtraction analysis, MKDA with logistic regression, parametric statistical tests, and parametric bootstrap method (with GPD tail-fitting). The activation maps are presented in Z-scores, showing regions with uncorrected *p*-values under 5% significance level.

We have previously demonstrated the consistency of activation regions detected by ALE and the CBMR parametric inference method for single-group CBMR analysis (Yu et al., 2024). In this section, we extend our investigation to datasets with multiple groups to assess whether this consistency persists, and to evaluate the similarity of activation regions identified by the CBMR inference using two methods for standard error estimation: Fisher information and a parametric bootstrap approach. Our analysis focuses on group-specific activation regions for each of the three groups and group-wise comparisons between any two groups in the Cue Reactivity dataset, which includes a total of 3, 197 foci (Hill-Bowen et al., 2021). As the total count of foci exceeds the practical recommendation of at least 200 foci per group, both the parametric statistical test and the parametric bootstrap test are plausible for this dataset. Therefore, we conduct the subsequent analysis using both methods to confirm the consistency of the findings. For comparison, we present uncorrected Z-statistic maps generated by the CBMR inference stage using ALE, the CBMR inference based on the parametric statistical tests described in Equation 12, and the parametric bootstrap method (with GPD tail-fitting) for all voxels significant at α=0.05 in Figures 5–8. To ensure comparable spatial resolution, we adopt the default FWHM determined by effect size in the Python package NiMARE (Salo et al., 2022).

Figures 5–7 demonstrate notable consistency in the detected activation regions (voxels with uncorrected significant *p*-values less than 0.05 ) across the three groups in the Cue Reactivity dataset. Similar consistency is also observed in the activation regions of the entire Cue Reactivity dataset, as reported in figures 5 and 6 of Yu et al. (2024). This consistency is particularly evident in the left cerebral cortex, frontal orbital cortex, insular cortex, and left and right accumbens. The observed activations in these regions during cue reactivity reflect the engagement of a complex neural network comprising multiple functional systems: reward processing and motivation, mediated by the nucleus accumbens and its dopaminergic projections; value-based decision making, supported by the orbitofrontal cortex, interoceptive awareness, and conscious craving mediated by the insula; cognitive control, attention and emotional regulations, associated with various parts of prefrontal cortex; and learning and memory processes, involving cue–outcome associations encoded in the hippocampus and amygdala. However, slight differences in spatial specificity and smoothness are observed between the methods: ALE provides the smoothest activation regions and detects the largest extent of activation, likely because it is sensitive to the spatial convergence of reported coordinates across studies rather than voxel-wise effect size magnitude or spatial specificity. In contrast, the parametric statistical tests and the parametric bootstrap method (with GPD tail-fitting) yield more stringent and localised activation regions. This is likely due to CBMR explicitly accounting for both within- and between-study variance, and often incorporates spatial heterogeneity and uncertainty more explicitly, leading to more conservative and spatial precise detection of significant voxels. Despite these differences, all methods exhibit high overall consistency, with only minimal differences in the location of detected activation regions.

**Fig. 9.**
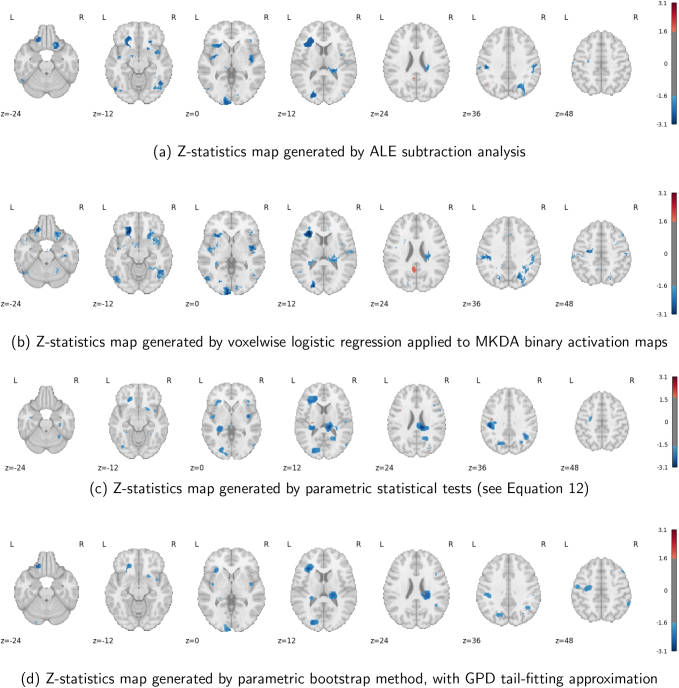
Difference in activation regions between the **Drug-Neutral** and **Reward-Neutral** groups: ALE subtraction analysis, MKDA with logistic regression, parametric statistical tests, and parametric bootstrap method (with GPD tail-fitting). The activation maps are presented in Z-scores, showing regions with uncorrected *p*-values under 5% significance level.

Figure 8 illustrates activation patterns observed in the group comparisons between the Drug and Natural groups within the Cue Reactivity dataset. Results from two additional group comparisons are presented in Figures 9 and 10 in Supplementary Appendix A.2.3. These figures highlight voxels with uncorrected significant *p*-values less than 0.05 , demonstrating the reliability of CBMR inference when applied to real datasets, in comparison with kernel-based methods. Brain regions highlighted in red (indicating positive *z*-statistics values and corresponding to uncorrected significant *p*-values) represent areas where one group shows stronger activation than the other group. Conversely, regions highlighted in blue (indicating negative *z*-statistic values and corresponding to uncorrected significant *p*-values) denote areas where the other group exhibits stronger activation. Figure 8 demonstrates strong consistency in findings across all four comparison methods (ALE subtraction analysis, voxel-wise logistic regression on MKDA activation maps, CBMR inference using either parametric statistical tests, or GPD tail-fitted bootstrap). This highlights the stability and accuracy of CBMR even in the presence of group size imbalance.

In the Cue Reactivity dataset, the (square root of) sample size and year of publication are considered as study-level covariates to understand their global effects on group-wise activation intensity functions. Our CBMR regression analysis indicates that the activation intensity function increases globally by 8.1587% for each unit increase in the square root of sample size, and decreases globally by 0.5397% for each unit decrease in the year of publication. We also conducted hypothesis testing to determine whether these two study-level covariates have a significant effect (i.e., whether their regression coefficients are distinguishable from zero). Under the null hypothesis that these study-level covariates have no effect, we reject the null hypothesis for the square root of sample size at the 0.05 confidence level ($p=1.1732\times 10^{-9}$ ). However, we could not reject the null hypothesis for the year of publication (p=0.6681 ). Additionally, leveraging the flexibility of the CBMR inference framework, we compared the effects of these two study-level covariates. Under the null hypothesis that the effect of year of publication is stronger than that of the (square root) of sample size, we rejected the null hypothesis at 0.05 confidence level ($p=5.1857\times 10^{-5}$ ).

#### 3.2.3 Computation time

The computation time for CBMR multi-group analysis varies significantly between the inference stage using parametric statistical tests (as described in Equation 12) and parametric bootstrap method at voxel level. For large datasets with a sufficient number of studies and foci, where the numerical singularity of the Fisher Information matrix is not a concern, parametric statistical tests are more computationally efficient. These tests allow for flexible homogeneity or group comparison analyses with only a single meta-regression stage. On an NVIDIA GTX 1080 Graphics Card, this approach takes approximately 30 minutes to complete. However, for datasets with an insufficient number of studies or foci (fewer than 200 foci per group), the parametric bootstrap method becomes necessary during the meta-inference stage. This method involves generating 1, 000 bootstrap samples, requiring repeated randomisation of foci locations or data re-generation and model refitting for each bootstrap sample, significantly increasing computational complexity. Despite implementing parallelisation to accelerate the process, running model refitting on five bootstrap samples in parallel on a single HPC cluster node with Intel(R) Xeon(R) Gold 6126 2.60 Hz processors takes approximately 40 minutes. Achieving a comparable computational time to the parametric statistical test method would require around 200 HPC nodes. However, access to HPC resources with such computational capacity is often limited. Therefore, we recommend avoiding the parametric bootstrap method whenever possible and using parametric statistical tests for more computationally efficient analysis. Although CBMR remains more sufficient than fully Bayesian approaches, the reliance on large-scale HPC resources for bootstrap inference constitutes a practical limitation for some applications.

## 4 Discussion and Conclusion

In this work, we have detailed and presented multi-group CBMR, a module implemented in the open-source Python package NiMARE, designed for performing meta-regression and meta-inference on coordinate-based meta-analytic fMRI datasets. The meta-regression framework incorporates a spatial model based on spline parametrisation, where a roughness penalty is applied to regularise the smoothness of the spline basis functions. The meta-regression stage fits a generalised linear model with either Poisson or Negative Binomial (NB) distribution at the voxel level, and accommodates study-level covariates such as sample size and year of publication. Our approach also provides two distinct inference frameworks based on the number of studies or foci in each group within the dataset: For datasets with a sufficient number of foci (above a threshold of 200 foci per group), we recommend a computationally efficient inference method based on parametric statistical tests at the voxel level. This approach is significantly more efficient than the previously proposed Bayesian spatial regression model, while having the flexibility and interpretability of hypothesis testing for either spatial homogeneity or group comparisons. For datasets with an insufficient number of studies or foci, we propose a parametric bootstrap method as alternative for more accurate inference. In this method, *p*-values are obtained by comparing observed values with the null bootstrap distribution. While inherently computationally intensive—requiring repeated randomisation of foci locations or re-generation of data for thousands of bootstrap samples—this approach is necessary to address numerical issues related to inverting singular Fisher Information matrices in small datasets. Through simulations on synthetic data under various experimental settings, we demonstrated that meta-inference outcomes based on parametric statistical tests are valid for datasets with a sufficient number of foci (high underlying intensity functions). However, for datasets with an insufficient number of foci (low underlying intensity functions), *p*-values tend to skew liberally. Despite these challenges, meta-inference outcomes based on parametric bootstrap method remain valid and accurate even under the most challenging simulation settings with insufficient foci. Using the Cue Reactivity dataset, we found that the NB model is the preferred stochastic model, as indicated by model comparisons via Likelihood Ratio Test (LRT). The Poisson model, in contrast, cannot explain over-dispersion observed in foci counts. Meanwhile, we also compare the activation regions identified by both ALE and CBMR approaches, utilising both parametric statistical tests or parametric bootstrap method. These comparisons validate the accuracy and robustness of CBMR inference framework, whether for spatial homogeneity or group comparisons.

There are a few limitations in our work. We employ the parametric bootstrap method for meta-inference on small datasets with insufficient foci count, which improves inference accuracy at the cost of increased computational time. In this approach, the null distribution of the test statistic is generated by resampling under the null hypothesis. While our integration of the Generalised Pareto Distribution (GPD) for tail approximation alleviates the precision ceiling of standard bootstrap resampling (1/​B for B samples), the overall procedure remains computationally intensive. This resource demand constrains the applicability of our framework to large-scale meta-analytic datasets or settings without access to high-performance computing. Future work will, therefore, focus on strategies to preserve accuracy while reducing computation burden. Potential directions include leveraging asymptotic distributional approximations (e.g., normal or chi-squared) to reduce the necessary number of bootstrap replications (Bickel & Freedman, 1981; Hall, 2013). However, such approximations must be carefully evaluated to avoid biases if the test statistic’s true distribution deviates from the assumed theoretical distribution. Another promising avenue is the use of data-driven alternatives (e.g., Gaussian or mixture distributions fitted to the observed data) for approximating non-standard null distributions in complex modelling scenarios.

**Fig. 10.**
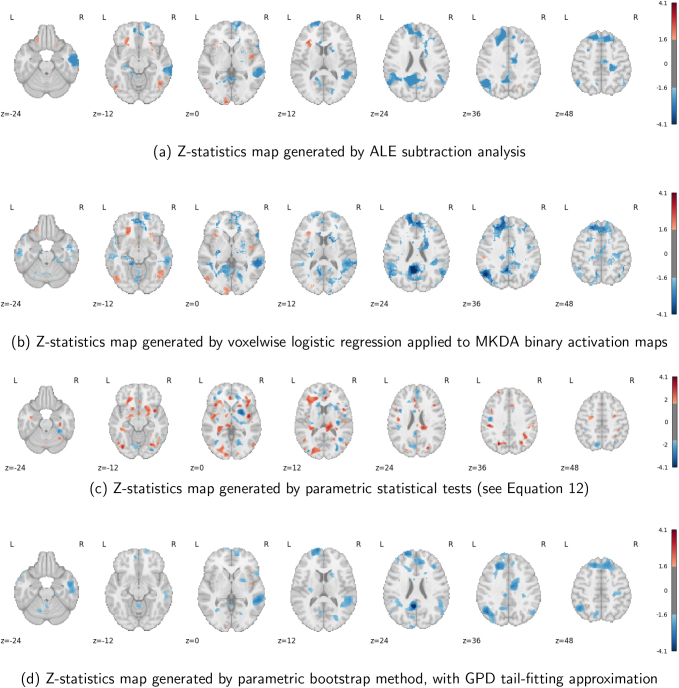
Difference in activation regions between the **Natural-Neutral** and **Reward-Neutral** groups: ALE subtraction analysis, MKDA with logistic regression, parametric statistical tests, and parametric bootstrap method (with GPD tail-fitting). The activation maps are presented in Z-scores, showing regions with uncorrected *p*-values under 5% significance level.

Another potential direction for future development is to ensure the CBMR framework more accessible to users without coding expertise or access to HPC clusters. Despite the significant effort invested in implementing CBMR as a module in the Python package NiMARE, its usage still requires basic coding knowledge, local environment setup, and familiarity with standard data preprocessing procedures. In contrast, platforms such as Neurosynth Compose (Kent et al., 2024) allow users to perform neuroimaging meta-analyses entirely within a browser, avoiding the setup for local Python environment. Neurosynth Compose allows users to search and integrate data from thousands of neuroimaging studies in the Neurosynth dataset and perform fast computations in the cloud using automated analysis pipeline. As a free and open platform for neuroimaging meta-analyses, it eliminates technical barriers for broader accessibility. Our next step is to integrate the CBMR regression and inference pipeline into the Neurosynth Compose platform. This integration will make CBMR accessible directly through a browser-based interface. Although CBMR inference via parametric bootstrap method is a valuable extension, particularly for studies with smaller sample sizes, it is also computationally intensive. In the absence of parallelisation or high-performance computing resources, bootstrap procedures can impose a substantial computational burden. Consequently, we do not plan to implement this functionality in Neurosynth Compose in the near future, as such demands would place impractical demands on the shared server infrastructure. Looking ahead, we aim to explore cloud-based solutions to accelerate the parametric bootstrap procedure, enabling efficient and scalable performance for computationally demanding analyses.

There is also significant potential for further theoretical development in conducting meta-analyses using data from multiple sources. With the growing convention among researchers to share full statistical maps, it is increasingly important to integrate additional information from both reported foci or full statistical maps (e.g., *p*-values or *t*-values), when available. Some researchers have proposed Markov melding as a fully Bay0065sian framework for joining probabilistic sub-models. In this method, evidence from different sources is specified in each sub-model, and sub-models are joined while preserving all information and uncertainty (Goudie et al., 2019). This approach could enhance inferences derived from CBMR by integrating the magnitude of CBMR activation or even data from image-based meta-analytic results. Another promising avenue for future development involves using CBMR inference outcomes as weights to determine the contribution of voxel-wise statistics from individual studies to the synthesised meta-analytic results. A well-designed choice of voxel-wise weights could stabilise variance and control heterogeneity by ensuring that studies with greater variability contribute less to the overall meta-analysis. Since CBMR inference outcomes involve voxel-wise variation for each study, they provide a data-driven approach for weighting. Future research will explore where these weights outperform existing methods based on inverse variance, sample size, or effect size.

## Data Availability

The code used in this work is available on https://github.com/yifan0330/Multi-group-CBMR. The code for analysis on the new dataset using the open-source Python package NiMARE is available at https://nimare.readthedocs.io/en/latest/generated/nimare.meta.cbmr.html. The authors do not have permission to share the data.
